# Linker Methylation as a Strategy to Enhance PROTAC Oral Bioavailability: Insights from Molecular Properties and Conformational Analysis

**DOI:** 10.1021/acs.jmedchem.5c01497

**Published:** 2025-08-01

**Authors:** Diego Garcia Jimenez, Giuseppe Ermondi, Zuzana Jandova, Maura Vallaro, Giulia Caron, Heribert Arnhof

**Affiliations:** 1 Molecular Biotechnology and Health Sciences Dept., 9314 Università di Torino, Piazza Nizza 44bis, Torino 10126, Italy; 2 33433 Boehringer Ingelheim RCV GmbH Co KG, Dr. Boehringer-Gasse 5-11, Vienna 1121, Austria

## Abstract

In this study, we profiled 11 structurally related von Hippel–Lindau (VHL)-based proteolysis-targeting chimeras (PROTACs), evaluating *in vivo* pharmacokinetics in mice (oral bioavailability and clearance), *in vitro* ADME properties (solubility, permeability, and efflux ratio), and key physicochemical traits (polarity, lipophilicity, and chameleonicity). While Caco-2 permeability did not correlate with oral bioavailability (F%), the efflux ratio (ER) proved a strong predictor. The ER could also be estimated using the chromatographic descriptor log k′80 PLRP-S. Conformational sampling and molecular dynamics in polar and nonpolar environments showed that linker methylation drives chameleonic folding, influencing ER and, in turn, F%. Overall, our results show that the oral bioavailability of VHL-based PROTACs with different linker methylation levels can be predicted throughout drug discovery. However, this requires specialized tools tailored to the challenges of PROTAC chemical space. Further work is needed to develop a robust, standardized, and automated predictive workflow.

## Graphical abstract

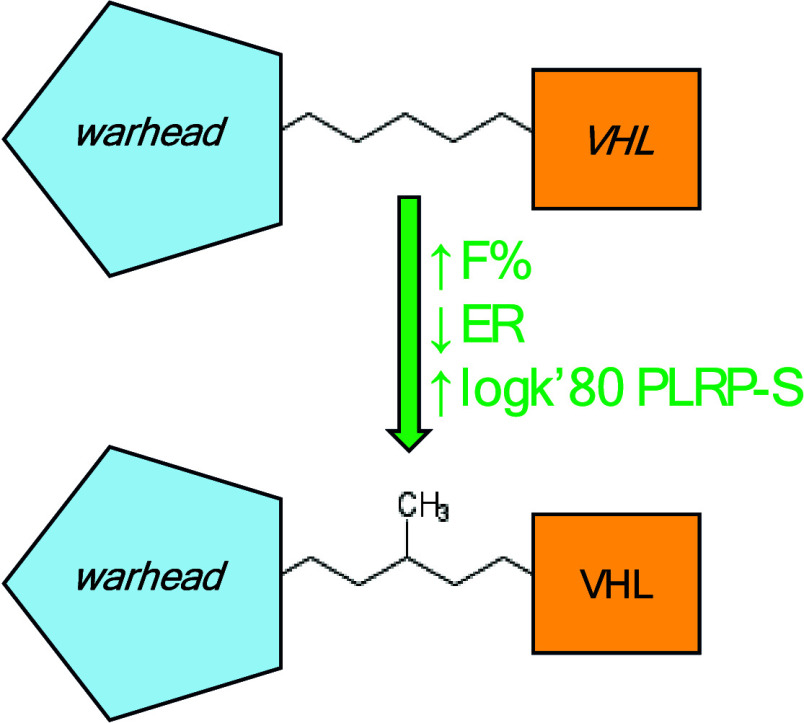

## 1 Introduction

The challenges and uncertainties associated with drug development for cancer and other unmet diseases call for innovative chemical modalities with novel mechanistic approaches.^1^ In this context, an emerging technology for selective protein degradation known as proteolysis-targeting chimeras (PROTACs) represents an attractive technology.^2,3^ PROTACs are heterobifunctional small molecules containing two ligands joined by a linker. The former ligand binds to the protein target of interest (POI) while the second ligand binds and recruits an E3 ubiquitin ligase (often CRBN or VHL), leading to the selective degradation of the POI via the ubiquitin-proteasome system.^4^ The structural and conformational complexity of PROTACs includes them in the beyond-rule-of 5 (bRo5) molecules.

It is widely accepted that oral administration for systemically acting drugs is the preferred route for its convenience, high patient compliance, and cost-effectiveness.^5^ Oral administration is characterized by absorption (drug entering the portal circulation) and bioavailability (F%, the fraction reaching systemic circulation after first-pass metabolism).^6,7^ High oral bioavailability can enable lower dosing and reduce pharmacokinetic variability, which may help minimize side effects and improve therapeutic consistency.^8^ In clinical trials, low bioavailability often results in drug candidate failure. Acceptable oral bioavailability for PROTACs was already verified in the literature, but issues with large and flexible structures, mostly associated with VHL derivatives, have been reported as well.^7,9,10^ In fact, despite an increasing number of orally dosed bifunctional degraders in the clinic, most disclosed to date rely on the CRBN E3 ligase recognition moiety.^11,12^

Experimental assessment of bioavailability is notably expensive and time-consuming. Consequently, alternative nonanimal approaches that align with the 3Rs principle are highly desirable.^13^ This highlights the need for strategies that can prioritize drug candidates based on their predicted oral bioavailability (F%) at various stages of the drug discovery process. To do so it is mandatory to know bioavailability determinants. Oral bioavailability is influenced by the molecular properties of the drug and the physiological characteristics of the organism, including metabolism (that is not considered in this study).^12,14^ The first category includes *in vitro* ADME (e.g., solubility and permeability) and physicochemical properties such as lipophilicity, polarity and more recently chameleonicity^14–17^ (i.e., the compound's capacity to modify their conformations and their properties to adapt to the different environments present in the human body). Therefore, once the chemical matter is available, experimental determination of *in vitro* ADME and physicochemical descriptors is widely utilized to assess and prioritize PROTAC drug candidates based on their predicted bioavailability (F%).

Furthermore, the medicinal chemistry community seeks to predict F% from scratch during very early drug discovery when chemical matter is absent, using computational tools such as physiological based pharmacokinetic modeling and simulation (PBPK), quantitative structure–property relationship (QSPR), machine learning algorithms and others (comprehensive review of these methods is outside the scope of this paper). To this respect, an emerging approach in the bRo5 space is based on assessing conformational variability in both polar and nonpolar environments. The strategy posits that a successful bRo5 candidate with acceptable oral bioavailability is likely to adopt open conformations in polar environments (e.g., GI tract, blood, plasma) and folded conformations in less polar environments (e.g., cell membrane interior). For instance, Vasanthanathan et al. demonstrated that more spherical and less polar conformations in chloroform enhance cell permeability in a series of CRBN-based PROTACs.^16,17^ However, only a few conformational studies on PROTACs have been published so far.^18–21^ Remarkably, in these studies the impact of the conformation in aqueous environments is often unexplored.^22^ Overall, no established criteria exist for prioritizing PROTACs based on F% modeling. This is partly due to the limited availability of public oral bioavailability data, routinely generated by pharma companies.^22,23^ Another reason for the relative lack of studies predicting F% from molecular properties also reflects the mechanistic complexity of F%, which includes first-pass hepatic metabolism and systemic clearance. As such, most modeling efforts have focused on absorption-driven parameters, which are more mechanistically aligned with these molecular properties.^24^

Recently, Kofink et al.^12^ have described the design of ACBI2 and have shown that small linker modifications influence compound conformations, leading to more compact arrangements with reduced 3D polar surface area and radius of gyration. They have also shown that more compact conformations may enhance absorption, reduce efflux, and ultimately improve oral bioavailability.

Starting from the aforementioned findings, this paper, the result of an academic–industrial collaboration, demonstrates how linker methylation can enhance the oral bioavailability of a series of VHL PROTACs using integrated experimental and computational approaches to assess F% across various stages of drug discovery. To reach this aim, we selected three series of SMARCA2/4 PROTAC with 11 compounds in total, representing diverse matched molecular series (MMS) (Figure 1a,b).

**Figure 1:**
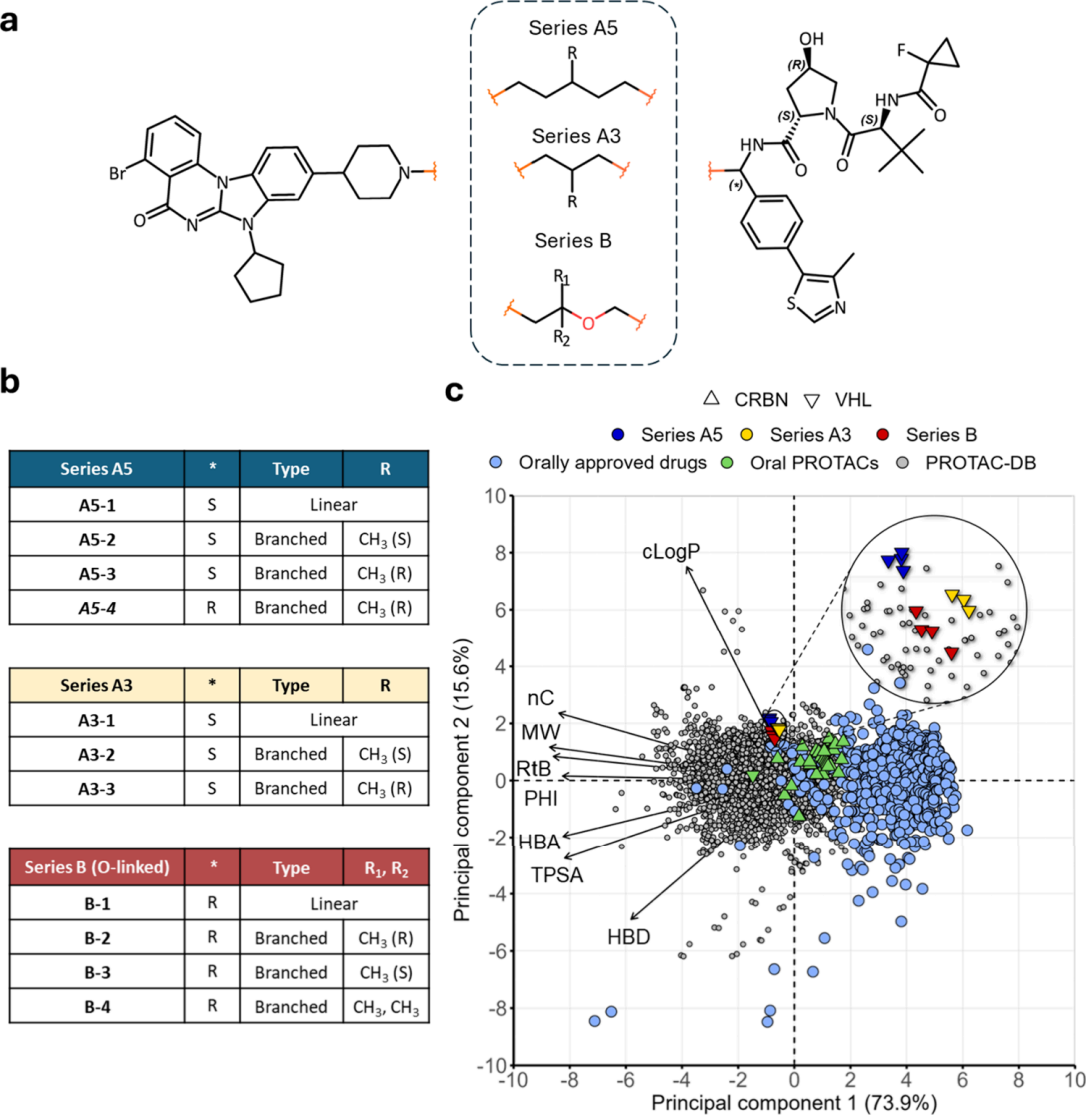
Studied PROTAC series. (a) Common chemical structure of the 11 studied PROTACs. The * symbol represents a variable stereochemistry in the attaching carbon to the 4-methyl-5-phenyl-1,3-thiazole. Calculated p*K*_a_'sfor the warhead's piperidine are in Table S1b. (b) Structural differences of the PROTAC data set. A5–4 is structurally related but does not belong to series A5 due to the different stereochemistry. (c) PCA representing the chemical space of the respective series compared to approved oral drugs, the PROTAC-DB, and orally available (F% > 5) published PROTACs.^7^ Compounds with MW > 1500 Da were removed from the plot. The length of the variables indicates their relative importance for PC1 and PC2.

These compounds degrade the SMARCA2 protein using a quinazoline-based scaffold acting as warhead and a VHL-based ligand to recruit the VHL E3 ligase.^12,25^ Structurally, they differ in the stereochemistry of the E3 ligand (marked as * in Figure 1) and in the linker, in terms of length, absence/presence of linker methylation, methylation stereoisomerism and absence/presence of polar atoms. In practice, the 11 PROTACs can be classified in three 3 series: series A5 and A3 display 5 and 3 carbons in the linker backbone, respectively, with further linker modifications depicted in Figure 1b. Series B has a different E3 ligand stereochemistry (*) and contains a linear 3-carbon backbone linker with an ether group. Notably, this series includes a dimethylated PROTAC. Linker methylation—the strategic addition of methyl groups to the linker—can be seen as a fine-tuning tool within “linkerology” since it may serve as a minimalist approach to achieving partial rigidification within a flexible linker.^26^ Notably, linker methylation was recently shown to profoundly affect the conformational and property landscape of macrocycles. This supports that for both macrocycles and PROTACs conformation dictates function.^27,28^

For the aforementioned 11 PROTACs, we compiled a comprehensive data set of experimental measurements, including *in vivo* oral bioavailability in mice, *in vitro* ADME properties, and a comprehensive pool of physicochemical descriptors. We then analyzed the relationship between bioavailability (F%) and its influencing factors. Finally, to rationalize how linker branching influences variations in F%, we utilized conformational sampling (CS) and molecular dynamics (MD) simulations to conduct a detailed molecular-level analysis in two distinct environments.

Overall, this work provides preliminary guidelines to prioritize linker methylated VHL-based PROTACs for their oral bioavailability at the different stages of the drug discovery process. While the study is robust, caution is warranted when extrapolating the findings to other scaffolds, particularly CRBN-based PROTACs.

## 2 Results

### 2.1 Chemical Space Encompassed by the Data Set

Recent studies emphasized the utility of chemical space analysis in assessing the relative position (molecular descriptor similarity) of any considered molecule compared to a set of reference compounds with known properties like bioavailability (F%) or permeability.^7,29–31^ In this study, we aim to position the 11 selected PROTACs within the chemical space defined by a) FDA-approved oral drugs, b) the PROTAC chemical space, and c) a collection of published oral PROTACs or those in clinical trials as of September 2023.^7,32^ To do that we calculated a set of 2D molecular descriptors for the investigated derivatives (Table S1). Figure 1c shows that the 11 PROTACs (blue, yellow and red triangles) occupy a space at the limit of the oral space (light blue dots). In particular, the three series occupy a region with higher molecular complexity (size, flexibility, polarity) than CRBN PROTACs (upward-pointing triangle). Furthermore, a comparison with PROTAC-DB, the most well-known database of PROTAC derivatives (gray dots), suggests that these 11 compounds are among the most lipophilic degraders (cLogP) reported. Notably, they exhibit higher lipophilicity than all other publicly available oral PROTACs (green dots). This observation suggests that achieving adequate oral bioavailability is likely to require a significant degree of chameleonicity. Moreover, all of them have a TPSA below 200 Å^2^, upper threshold for oral absorption suggested by Hornberger and Araujo, from a set of around 1800 degraders.^6^

### 2.2 Experimental Data

All the experimental data are in Table 1 and discussed below.

**Table 1:**
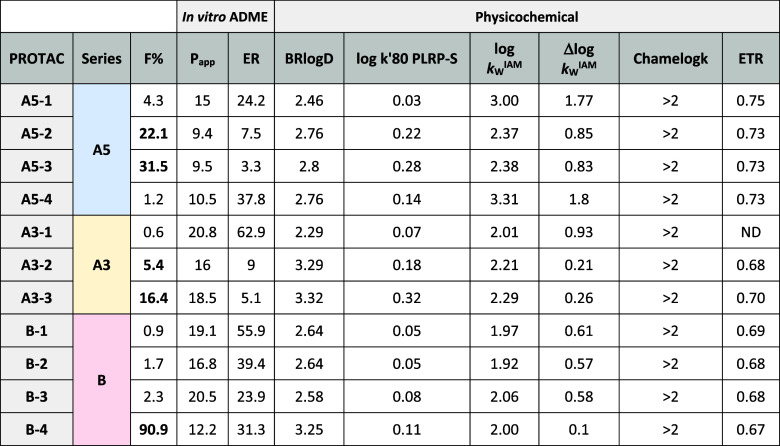
Experimental ADME and Physicochemical Descriptors for the 11 PROTACs^a^,^b^,^c^,^d^

a F% is the oral bioavailability in mice (high values are highlighted in bold, F% > 5).

b Passive permeability (*P*_app_ × 10^‑6^cm/s, Caco-2 assay in the presence of a P-glycoprotein inhibitor) is measured as the mean of the apical-basal (AB) and basal-apical (BA); the ER is the Caco-2 efflux ratio in the absence of an inhibitor.

c The physicochemical data include three lipophilicity descriptors (BRlogD, log k′80 PLRP-S, and log *k*_w_^IAM^, their description is available in the Methods section), one polarity determinant (Δlog *k*_W_^IAM^), one chameleonic index (Chamelogk), and the ETR descriptor (EPSA-to-TPSA ratio).^33^

d Additional data are reported in Table S2.

Bioavailability in mice was determined by comparing plasma exposure after oral administration with plasma exposure after intravenous administration (see Experimental Part). The investigated data set includes PROTACs with very low (*F* < 1%), low (*F* < 5%) and medium/high (*F* > 5%, in bold in Table 1) oral bioavailability.^7^ Only low-clearance PROTACs were investigated, with clearance of less than 10% of the hepatic blood flow, to avoid extensive hepatic first-pass effect (Mouse CL see Table S2). Notably, F% increases with the presence of methyl groups in any series.

Aqueous solubility was determined from a concentrated DMSO stock diluted into aqueous media. The soluble part was analyzed after 24h incubation. All the considered PROTACs are soluble enough to guarantee the reliability of permeability determination. Permeability (expressed as the arithmetic mean of *P*_app_ (AB) and *P*_app_ (BA) in the presence of a P-gp inhibitor, see Experimental Part) and efflux ratio (expressed as ER) were measured using Caco-2 cells. All the considered PROTACs are cell permeable (*P*_app_ > 1*10–6 cm/s) and show a remarkable ER. Notably, within the series, the presence of methyl groups does not enhance permeability but reduces the efflux ratio (ER).

Molecular properties have been quantified with physicochemical descriptors of ionization, lipophilicity, polarity and chameleonicity (see Methods). First, ionization of the 11 PROTACs (basic center, Figure 1a) was analyzed by potentiometry.^34^ However, standard p*K*_a_ measurements in water were limited by solubility and precipitation issues (data not shown). Therefore, to assess the dominant species at physiological pH we adopted a chromatographic method previously published by some of us.^35^ Briefly, we monitored the trend of the capacity factor named log k′80 PLRP-S at different pHs (acid-neutral-basic). The pH-dependent retention profile in the PLRP-S system revealed that at pH = 7.0 the compounds are mostly neutral and only partially ionized (Figure S1). The most neutral feature of the considered PROTACs in in line with the main role played by the neutral species in crossing membranes.^36^

Lipophilicity was assessed using three chromatographic descriptors, each simulating distinct aspects of membrane environments. BRlogD is a surrogate of the octanol/water distribution coefficient (log *D*_oct_) reflecting a general partitioning across biological membranes.^37^ Log *k*_W_^IAM^ implements an Immobilized Artificial Membrane (IAM) column which provides a lipophilicity index in an environment resembling the membrane phospholipids.^38^ Finally, log k′80 PLRP-S reflects retention in a hydrophobic polymeric column environment, mimicking the nonpolar core of lipid bilayers, and acts as a surrogate for the toluene/water partition coefficient (log *D*_tol_).^35^ Lipophilicity remained within a moderate range across all systems.

Polarity was quantified by Δlog *k*_W_^IAM^, a descriptor obtained from the experimental log *k*_W_^IAM^ and BRlogD.^39^ In addition, we also measured EPSA (Table S2), another polarity descriptor based on a supercritical fluid chromatographic (SFC) system widely used in drug discovery.^40^ EPSA indicated high polarity across the entire data set (>120 A^2^), while Δlog *k*_W_^IAM^ revealed excessive polarity (>1.5) only for A5–1 and A5–4. Notably, a moderate correlation was found between EPSA and Δlog *k*_W_^IAM^ (*R* ^2^ = 0.53, Figure S2).

Methylation affects lipophilicity and polarity differently across the series. In series A3, it consistently increases lipophilicity and decreases polarity, following expected trends. In series A5, methylation unexpectedly reduces IAM lipophilicity while showing trends similar to A3 for other descriptors. In series B, single methylation does not have a clear impact on lipophilicity or polarity (unexpected), but demethylation leads to increased lipophilicity (primarily BRlogD) and decreased polarity.

Chameleonicity was monitored by Chamelogk, a recent chromatographic descriptor able to capture environment-dependent conformational changes by monitoring different mobile phase compositions on the PLRP-S column.^15^ All PROTACs behave as strong chameleons (Chamelogk > 0.6).^15^ Additionally, we calculated the EPSA-to-TPSA ratio (ETR), a recently proposed index for polarity masking. The ETR values remained consistently around 0.7 across the series, supporting the notion that conformational effects contribute uniformly to the polarity profiles of the analyzed PROTACs.^33^

Overall, the physicochemical data confirm that the studied PROTACs emerged from a refined Hit-to-Lead phase, during which they were optimized to align with key design principles. These include intermediate-to-high lipophilicity, controlled polarity, and high chameleonicity, ensuring a balance of essential molecular properties.

As highlighted in the Introduction, predicting the likelihood of PROTACs to achieve oral bioavailability is essential across multiple stages of the drug discovery process. Therefore, we first monitored the impact of *in vitro* ADME descriptors on F%. For the investigated series of PROTACs passive permeability showed no relationship with F% (Figure S3). Conversely, the efflux ratio (ER) was shown to highly impact PROTAC F% (Figure 2a). In practice, PROTACs that are substrates of P-gp are poorly oral bioavailable.

**Figure 2:**
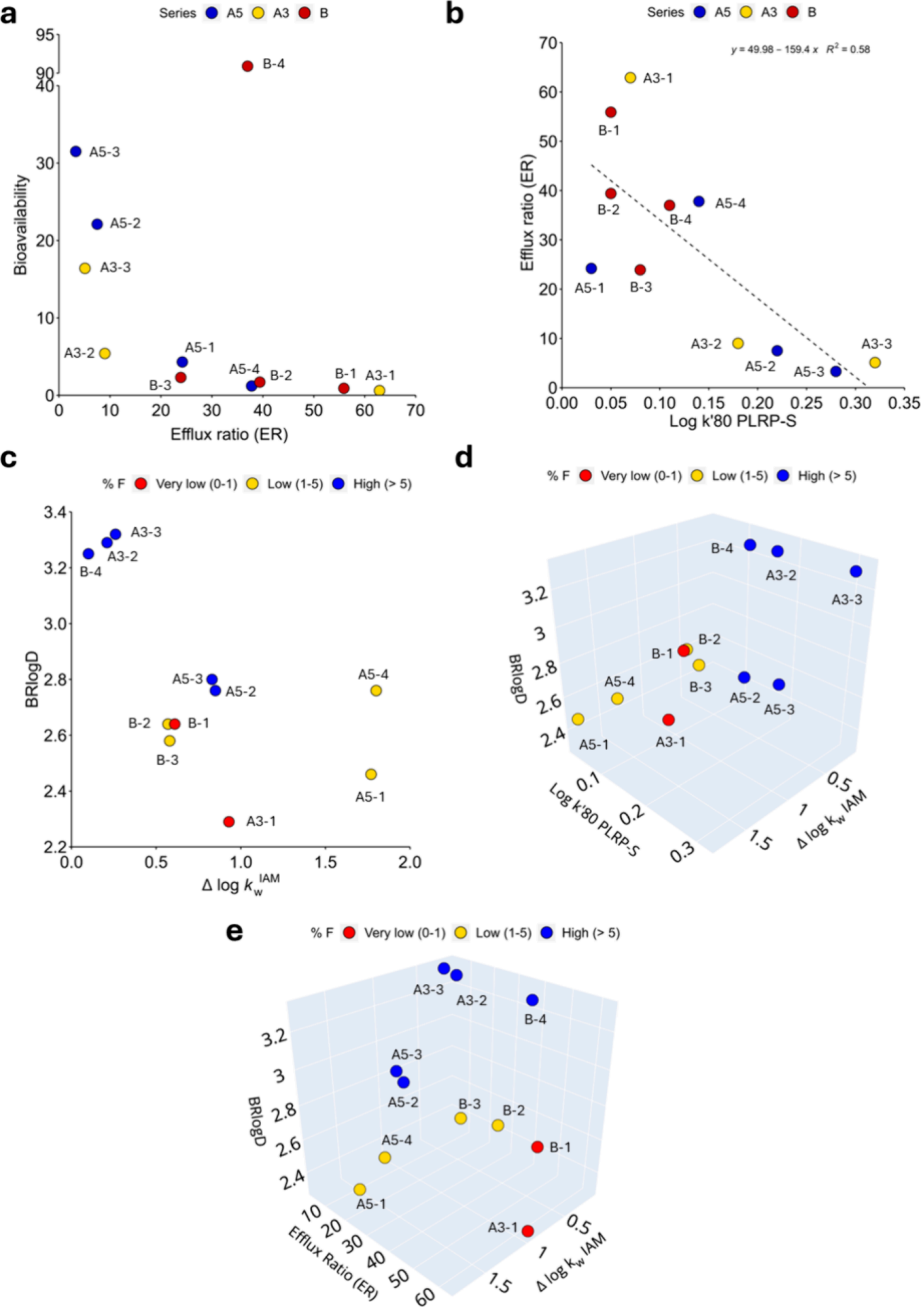
Correlations between physicochemical, *in vitro*, and *in vivo* ADME descriptors. (a) Efflux ratio vs F%. (b) Log k′80 PLRP-S vs efflux ratio. PROTAC F% explained by (c) BRlogD and Δlog *k*_W_^IAM^, (d) BRlogD, Δlog *k*_W_^IAM^, and log k′80 PLRP-S, and (e) BRlogD, Δlog *k*_W_^IAM^, and ER. B-4 is considered an outlier in plot (b).

Next, we explored the relationship between F% and ER with physicochemical descriptors. First, we verified that F% was not strongly correlated with any single descriptor. Remarkably, ER displayed a significant inverse correlation with log k′80 PLRP-S (*r* ^2^ = 0.58, Figure 2b). Since this latter is a membrane core partitioning surrogate,this result is in line with the literature reporting that the P-gp captures more its substrates in the hydrophobic core of the membrane than in the aqueous environment outside the cell membrane (flippase/pump models, respectively).^41,42^ Moreover, the literature also suggests that low polarity favors ER evasion^43^

In recent publications we observed how the balance between polarity (Δlog *k*_W_^IAM^) and lipophilicity (BRlogD) can help identify zones of higher oral absorption.^15,21,44^ Applying this descriptive approach to our data set revealed how the 5 PROTACs with the highest F% (>5) sit in a region of high lipophilicity and low polarity (blue dots in Figure 2c and Figure S4).^34^ Moreover, F% was effectively explained by using three coordinates, which included lipophilicity in the inner core of membranes (log k′80 PLRP-S), along with BRlogD and Δlog *k*_W_^IAM^ as shown in Figure 2d). Finally, the use of ER along with BRlogD, Δlog *k*_W_^IAM^ seemed to better discriminate between low and very low F% PROTACs (Figure 2e).

When examining the series separately, Figure 2 confirms that the effect of methylation varies depending on the series under consideration. In Series A5 and A3, we observed that adding a methyl group (either R or S configuration) decreases polarity and increases lipophilicity compared to the unmethylated derivatives, resulting in higher F% values. In Series B, however, the unmethylated and single-methylated derivatives exhibit similar behavior in lipophilicity and polarity terms. Adding a second methyl group (B-4), though, increases lipophilicity and reduces polarity, which enhances F% values.

Overall, the experimental data confirms that the efflux ratio plays a crucial role in determining oral bioavailability, with its impact being driven by specific molecular properties.

### 2.3 Chameleonicity and Conformational Effects Induced by Branching Linkers Explain Variations in Oral Bioavailability

Below we discuss how *ad hoc in silico* simulations may help identify conformational effects modulating oral bioavailability. PROTAC A5–4 was excluded from further analysis because of the different stereochemistry of its E3 ligand (S to R).

#### 2.3.1 Chameleonicity

Chameleonicity is typically linked to the adoption of folded, less polar conformations in nonpolar solvents, and extended conformations in water. It can be assessed through conformational sampling (CS) in implicit solvent analysis. Specifically, the conformers generated from simulations are characterized by their polarity, size, and shape behavior.^20^ In this study, the molecular polar surface area, and referred to as 3D PSA 0 Å, was chosen as the polarity descriptor. The radius of gyration (*R*_gyr_) was employed to assess the sphericity of PROTACs. As previously discussed in other studies, all the PROTACs analyzed exhibited chameleonic behavior, with polarity distinguishing conformers in chloroform (less polar) from those in water (more polar) (Figure 3).^18,20,21,45^

**Figure 3:**
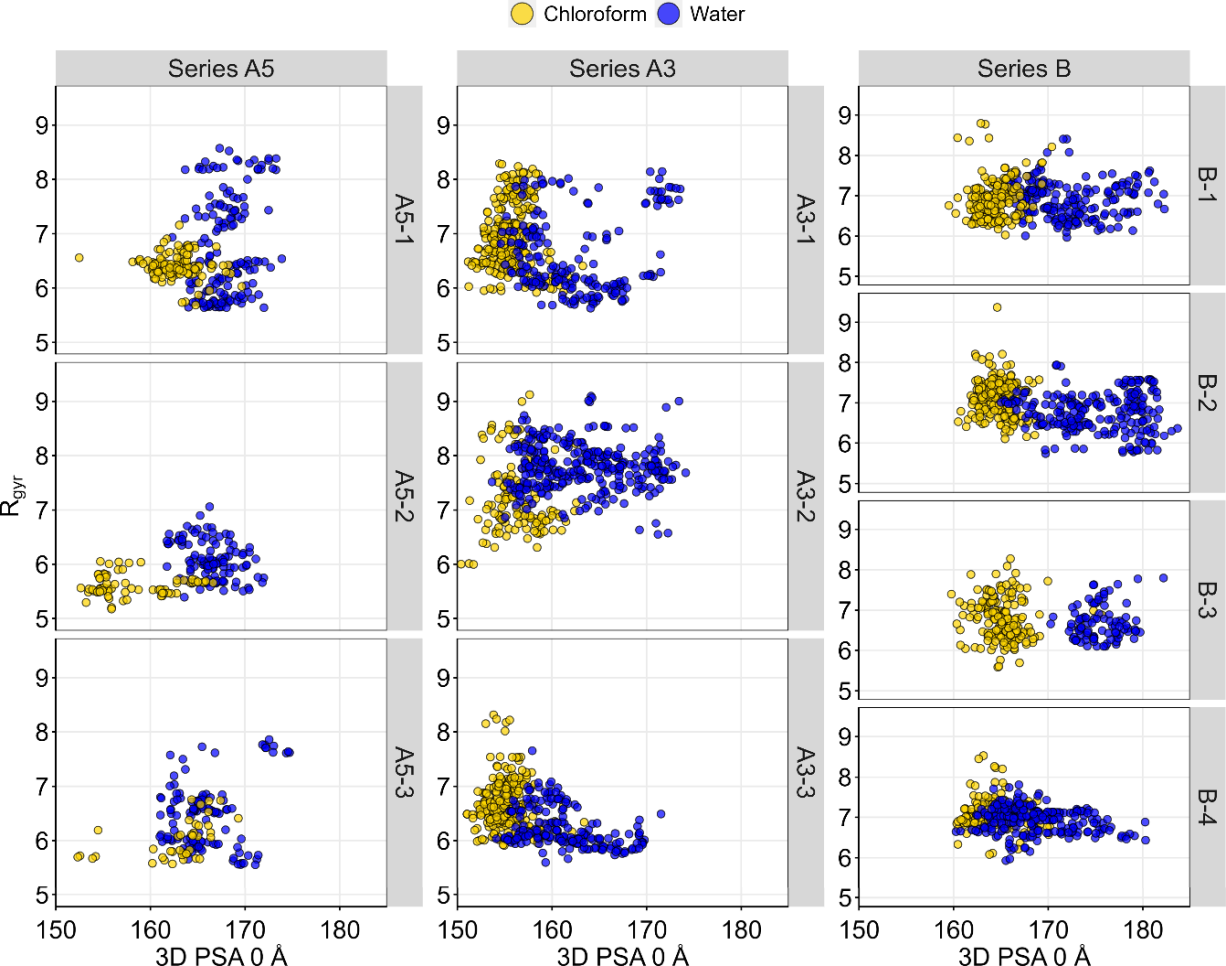
Plots of radius of gyration (*R*_gyr_) versus 3D polar surface area (3D PSA at 0 Å) for conformational ensembles of the studied PROTACs, generated through conformational sampling. Data points are color-coded according to the implicit solvent used in the simulations.

In an earlier study, some of us demonstrated through simulations that chameleonicity in a series of structurally related CRBN PROTACs is driven by specific intramolecular hydrogen bond (IMHB) patterns.^21^ However, conformational sampling for these PROTACs did not indicate any IMHB formation (data not shown), ruling out IMHB as a chameleonicity driver in this data set.

#### 2.3.2 Shape and Polarity Variations Induced by Branching Linker

Visual inspection of the CS profiles (Figure 3) roughly shows that in water, the range of *R*_gyr_ decreases as methylation increases. Additionally, experimental data (as discussed earlier) supports that methylation affects ER variation within the PROTAC series. To further explore these findings, we performed 20 ns steered molecular dynamics (SMD) simulations in explicit solvent in two different media (Figure 4). We then analyzed the results using density maps, which represent the conformer population density across a bidimensional axis (i.e., 3D PSA 1.4 Å and *R*_gyr_).

**Figure 4:**
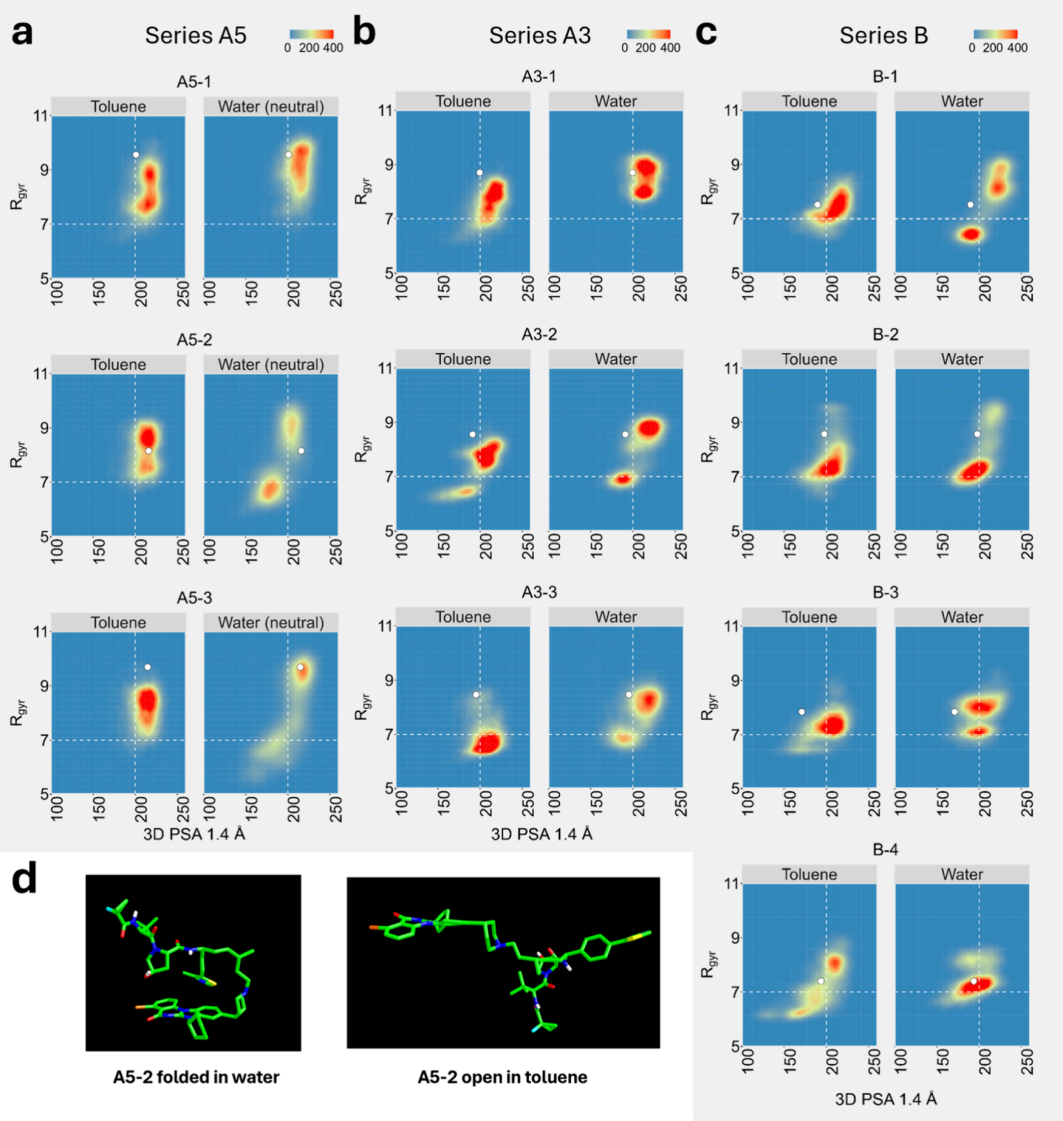
SMD-derived property space analysis. (a–c) Conformer density plot for series A5, A3, and B, respectively, faceted by solvents: toluene and water. Color coding represents the population density in each plot region. White dots represent the starting conformations of the SMD simulations. Horizontal and vertical lines at *R*_gyr_ = 7 and 3D PSA 1.4 Å = 200 Å^2^, respectively, are manually placed to aid in the PROTAC space comparison. (d) Conformers representing the density maximum for A5–2 in water and toluene. Violin plots for all series divided by solvents (*R*_gyr_ and 3D PSA) are found in Figure S5 *.*

In Series A5, compound A5–1, which features a linear linker, along with A5–2 and A5–3 (which contain a methyl group in the linker in the S and R configurations, respectively), exhibit similar shape profiles (*R*_gyr_) in toluene (Figure 4a). However, in water, the methylated compounds tend to adopt more spherical conformations (low *R*_gyr_) and display lower polarity (Figure 4a,e,f). In water, the methyl group in both A5–2 and A5–3 is exposed to the solvent, allowing the warhead and E3 ligand to fold (as shown in Figure 4d, where the two conformers representing the density maximum for A5–2 in both water and toluene are depicted). In contrast, A5–1 remains fully extended in both solvents. To further investigate this, we conducted longer SMD simulations (150 ns) and observed that the PROTACs with the methyl group fully fold in aqueous environments (Figure S6). Additionally, 20 ns SMD runs were performed starting from spherical conformations (*R*_gyr_ = 7) (Figure S7), and the resulting property space confirmed these findings. These results demonstrate that the presence of a methyl group in a 5-carbon linker can significantly alter the conformational space of the PROTAC.

Series A3 includes one candidate (A3–1) with a linear linker derivative and two others, A3–2 and A3–3, with methylated linkers. In water the absence of a methyl group in the alkylic linker (A3–1) prevents the folding. The reverse is true for the two methylated derivatives (Figure 4b,e,f) which display folded, low polarity conformations. Moreover, A3–2, tends to fold into more folded conformations also in toluene. A3–3 follows the same trend as A3–2 but to an even greater extent. Overall, as with series A5, the presence of a methyl group in the alkyl linker of the A3 series also appears essential for achieving folded conformations (Figure 4).

Series B includes four PROTACs bearing an oxygenated linker (Figure 1). The PROTACs differ by the presence of zero, one (R or S) or two methyl groups in the linker structure. At first sight, SMD revealed that structural modifications of the linker do not have a strong impact on the polarity and shape/size property landscape of this series (Figure 4c). All molecules seem to be partially folded in water and toluene. The only exception is B-4, which contains two methyl groups and shows in toluene a high propensity to fold and decrease polarity (Figure 4c,e,f). Notably, despite containing an oxygen group, this series tends to exhibit lower exposed polarity (3D PSA 1.4 Å) compared to the other series (Figure 4f). These results suggest that the oxygen is not exposed to the solvent.

Notably, PROTAC folding in aqueous media was also recently reported by Kihlberg and co-workers who studied two VHL PROTACs that differ only by the replacement of two methylene groups in the linker by oxygen atoms but that displayed vast differences in their cell permeability.^45^ Moreover, experimentally determined PROTAC structures—such as the one reported by Geiger et al.—have revealed cases where the PROTAC adopts a collapsed conformation, resulting in pronounced anticooperativity.^46^

#### 2.3.3 Conformational Profiles Offer a Molecular Explanation for the Experimental Data

To explore the relationships between MD simulations and experimental *in vitro* and *in vivo* data (see below) we defined numerical quantifiers describing MD results discussed above. We decided to use the median values of *R*_gyr_ and 3D PSA 1.4 Å, since they represent the most suitable statistical descriptors to keep track of the overall behavior of the simulation (Figure S5).

Figure 5a illustrates how computational descriptors help rationalize oral bioavailability data. In water, high globularity (low *R*_gyr_) and low polarity (low 3D PSA 1.4 Å) contribute to enhanced oral bioavailability (blue) across all series. In toluene, reducing polarity is always essential for improving bioavailability, but the impact of *R*_gyr_ variation is less significant.

**Figure 5:**
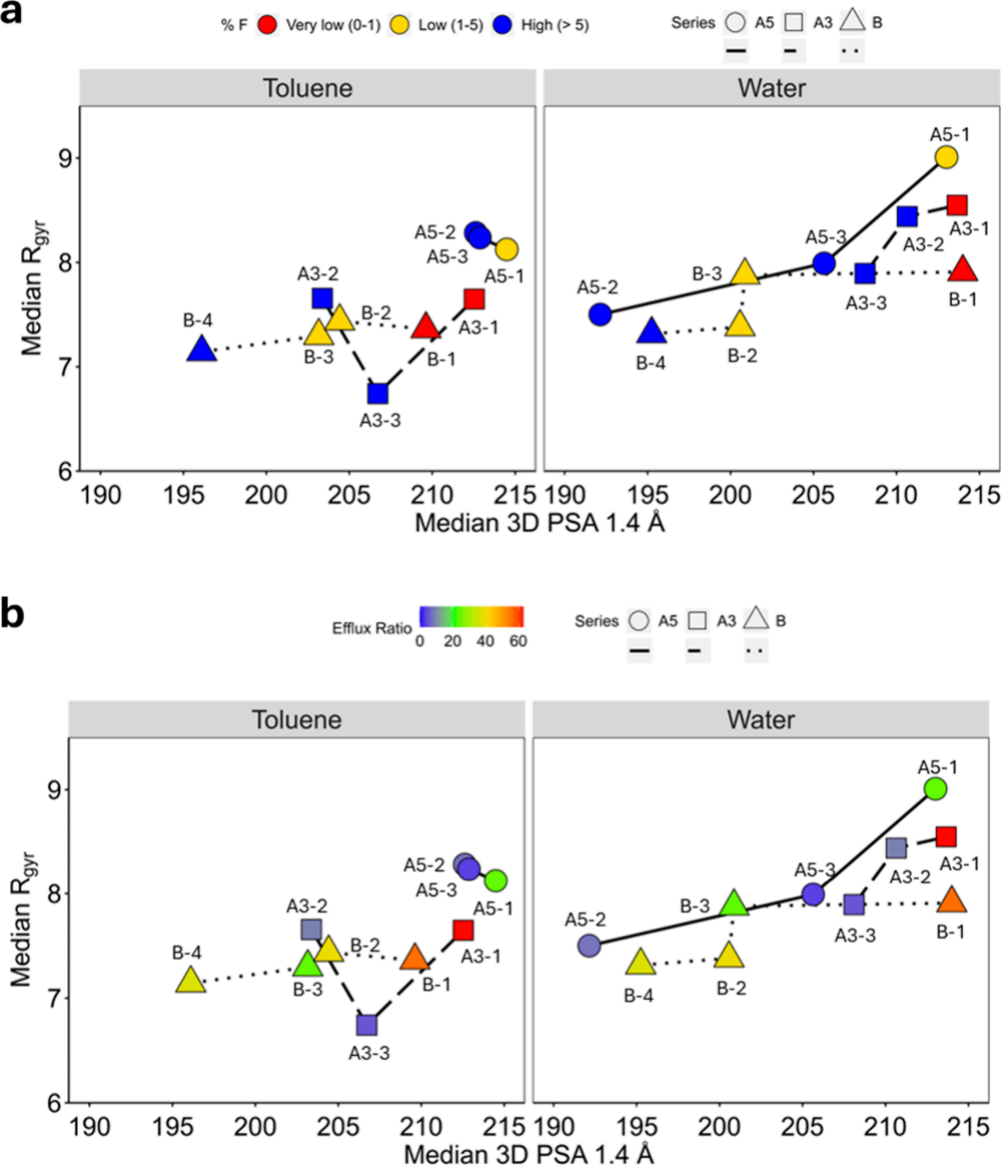
Capacity of 3D PSA 1.4 Å and *R*_gyr_ to explain the (a) F% and (b) ER for 20 ns SMD-derived conformation population distributions. Shapes and line-type styles are selected to distinguish between the series. The expanded figures faceted by the series are available in Figures S8 and S9. Unaggregated values are reported as part of Figure S5.

Overall, PROTACs with the highest F% are folded and exhibit low polarity in aqueous environments. Figure 5b provides additional insights, focusing on efflux ratio (ER) data. Notably, within each series, higher 3D polarity correlates with a higher ER. Furthermore, our data suggest that P-gp-mediated efflux is limited for PROTACs that adopt more folded conformations in aqueous media.

Figure 5a,b also demonstrate that the presence of oxygen in the linker (Series B) does not increase polarity, as indicated by experimental data (Table 1), though this is not reflected by TPSA. In fact, PROTACs in Series B exhibit lower exposed polarity (3D PSA 1.4 Å) compared to other PROTAC series in both environments (Figure 5). These findings are somewhat unexpected and suggest that the presence of methyl groups may effectively mask the polar effect of the oxygen.

## 3 Discussion and Conclusions

The primary conclusion of this study is that oral bioavailability (F%) of the 11 highly optimized SMARCA2/4 VHL-targeting PROTACs can be predicted in the early stages of drug discovery. This represents a significant advancement in the predictive modeling of VHL-based PROTAC pharmacokinetics, providing valuable insights for optimizing PROTAC development in the early phases of drug discovery.

A key finding is that F% is influenced by the efflux ratio (ER), not passive permeability, with ER measured through permeability assays. This highlights once more the importance of data quality in drug discovery and the need of assay modification to take into PROTACs peculiar structural properties.^24^ Cui et al. but also Muschong et al. demonstrated that compounds exceeding the Rule of 5 require assay modifications to address low recovery.^47,48^ While our results are solid, it remains uncertain whether P-glycoprotein expression in Caco-2 cells accurately reflects expression in cellular systems used for degradation activity. This suggests the option of also quantifying ER in cellular systems where degradation is assessed.

This study demonstrates that linker methylation is an innovative strategy for the property-based optimization of PROTACs, offering a new avenue for enhancing their pharmacokinetic properties. Beyond its role in improving physicochemical attributes, linker methylation could also serve at least in theory as a critical factor in stabilizing the formation of ternary complexes, which consist of the E3 ligase, the PROTAC, and the target protein of interest.^49^ These complexes often require the PROTAC to adopt specific folded conformations for efficient binding and functional activity. Therefore, methylation may not only optimize the PROTAC’s molecular properties profile but also facilitate the proper structural arrangement needed for effective target engagement. However, methylation effects are often context-dependent and may not consistently induce the desired rigidity or improve ternary complex stability across different scaffolds. As an alternative, rigid (spiro)­cyclic linkers offer conformational control, which may enhance cooperativity and selectivity in ternary complex formation.

This paper also shows that a set of physicochemical descriptors can effectively distinguish degraders with varying bioavailability. A recent literature review highlights the limited use of physicochemical descriptors in PROTAC drug discovery campaigns.^14^ Additionally, only ChromlogD and EPSA are commonly employed in pharmaceutical companies. This study underscores that relying solely on these two descriptors is insufficient for establishing robust property-based drug discovery strategies. For example, ER can be modeled using a lipophilicity descriptor like log k′80 PLRP-S, which is often overlooked despite its simplicity. Furthermore, log *k*_w_^IAM^ identifies PROTACs with a greater propensity to fold in water. It is also important to note that, as physicochemical data can be measured by automated systems, the increasing capabilities of AI/ML algorithms to model ADME data could lead to a new approach for monitoring F% based on the prediction of physicochemical properties.

In this study, the conformational variability of PROTACs is explored using both conformational sampling and molecular dynamics techniques, which offer distinct yet complementary insights. This represents a significant advancement toward establishing standardized computational protocols for their integration into industrial drug discovery pipelines.

Overall, the paper proves that F% of series of VHL PROTACs can be predicted at the different stages of the drug discovery process. To do so, the adoption of specific tools tailored to the PROTAC bRo5 space is mandatory. However, it is important to note that the absence of F% data for less-optimized PROTACs limits the generalizability of these conclusions. As a result, further studies are necessary to determine whether the predictive framework holds for compounds with lower levels of optimization.

## 4 Methods

### 4.1 Experimental Section

#### 4.1.1 *In* *Vivo* Oral Bioavailability

The authors confirm that the research in this study complies with all relevant ethical regulations. All animal studies were approved by the internal ethics committee (called “ethics committee”) of Boehringer Ingelheim RCV GmbH & Co KG in the department of Cancer Pharmacology and Disease Positioning. Furthermore, all protocols were approved by the Austrian governmental committee (MA 60 Veterinary office; approval numbers GZ: 903122/2017/21, GZ: 154399/2018/16, GZ: MA 58–670393–2019–18, GZ: MA 58–546776–2022–13). Female BomTac:NMRI-Foxn1nu mice were obtained from Taconic Denmark at an age of 6–8 weeks. After arrival at the local AAALAC accredited animal facility at Boehringer Ingelheim RCV GmbH & Co KG mice were allowed to adjust to housing conditions for at least 5 days before the start of the experiment, i.e. mice in all experiments were 7–9 weeks old. Mice were group-housed under pathogen-free and controlled environmental conditions (21 ± 1.5 °C temperature, 55 ± 10% humidity) and handled according to the institutional, governmental and European Union guidelines (Austrian Animal Protection Laws, GV SOLAS and FELASA). Animal studies were approved as described in the Ethics section. Food and water were provided ad libitum.

All pharmacokinetic studies were conducted with three mice per dose group and testing route. Oral bioavailability was determined by comparing the dose-normalized area under the plasma concentration–time curve (AUC_DN) following oral administration to the AUC_DN after intravenous (IV) administration of the same drug. All oral administrations were conducted with a volume of application of 10 mL/kg, typically at a dose of 30 mg/kg as a solution in a 25% to 40% HP-β-CD at a pH range of 4 to 5. Intravenous administrations were conducted at a dose of 5 mg/kg, with a volume of application of 5 mL/kg in 25% HP-β-CD at a pH of 5. Blood samples were obtained from the vena saphena at five time points after each administration, with the aim of measuring the drug concentration in the plasma. The concentrations of the compound in plasma samples were determined by quantitative HPLC-MS/MS, employing an internal standard. Calibration and quality control samples were prepared using blank plasma from untreated animals. The samples were precipitated with acetonitrile and subsequently injected into an HPLC system (Agilent 1200). The separation was conducted using a gradient of 5 mmol/L ammonium acetate at pH 4.0 and acetonitrile with 0.1% formic acid on a 2.1 mm by 50 mm XBridge BEH C18 reversed-phase column with 2.5 μm particles (Waters). The HPLC was interfaced with an ESI-operated triple quadrupole mass spectrometer (5000 or 6500+ Triple Quad System, SCIEX) operated in multiple reaction monitoring mode. The chromatograms were analyzed using Analyst (SCIEX), and the pharmacokinetic parameters, such as AUC_DN, were calculated using noncompartmental analysis with BI-proprietary software.

The oral bioavailability (F%) was calculated using the following formula: F% = AUC_DN_oral/AUC_DN_IV) * 100.

#### 4.1.2 *In Vitro* ADME

##### 4.1.2.1 Solubility

Compound solubility was determined by dilution of a 10 mM compound solution in DMSO into buffer to a final concentration of 125 μg/mL. Dilution into a 1:1 mixture of acetonitrile and water was used as reference. After 24h, the incubations were filtrated, and the filtrate was analyzed by LC-UV.

##### 4.1.2.2 Permeability, Efflux Ratio

Permeability and efflux ratio was derived by a bidirectional permeability measurements with Caco-2 cells. A modified protocol was used with a 24h preincubation of a 1 μM solution before the actual assay was started as described by Cui at al.^47^ For permeability and efflux ratio in the presence of a P-gp inhibitor, Zosuquidar was added throughout the experiment.

#### 4.1.3 Physicochemical Characterization

##### 4.1.3.1 Apparatus, Reagents and Methods

All chromatographic measurements were performed using the Thermo Scientific Inc. HPLC DIONEX Ultimate 3000 connected to an RS diode array and the Chromeleon 7.2.10 software (www.thermofisher.com). 1.5 mL HPLC vials, 9 mm PP screw caps, and high-performance ergonomic single-channel variable volume pipettors from VWR Signature were used. A Eutech pH Meter 2700 was used to regulate the pH of each buffer and sample (www.fishersci.com). For all the chromatographic descriptors, the mobile phase consisted of an acetonitrile solution (ACN) and 20 mM AAB, pH 7. Moreover, 10 μL was set as the volume of injection of compound concentrations ranging from 50 to 100 μg/mL in ACN/buffer mixtures. One mL/min was chosen as the isocratic flow rate and 30 °C as the oven temperature. However, each chromatographic system (RP) had its own conditions:

##### 4.1.3.2 BRlogD

BRlogD as an analog of log P_oct_ required the use of the XBridge Shield RP18 (130 Å, 5 μm, 5 cm × 4.6 mm) column by Waters (www.waters.com). The eluent was composed of a 60% ACN and 40% 20 mM ammonium acetate buffer (v/v) mixture. The retention time was measured for the analyte and the log k′60 was calculated (capacity factor k′60 = [*t*_R_ (60% ACN) – *t*_0_]/*t*_0_) and then converted to the corresponding BRlogD value using the equation BRlogD = 3.31*x* + 2.79. In addition, acetone, caffeine, ibuprofen, lidocaine, phenol, and a mixture of uracile, acetophenone, and toluene were used as gold standards.^35^

##### 4.1.3.3 Log k′80 PLRP-S

Log k′80 PLRP-S and Chamelogk, on the other hand, were measured using the PLRP-S polymeric reversed-phase column (100 Å, 5 μm, 50 × 4.6 mm) from Agilent (www.agilent.com). The assessment of log k′80 PLRP-S involved the measurement and calculation of the capacity factor of each sample at an eluent composition of 80% ACN and 20% 20 mM ammonium acetate at pH 7.0.^35^ In addition, acetone, caffeine, phenol and a mixture of uracile, acetophenone and toluene were used as gold standards. Moreover, log k′80 PLRP-S was also assessed at different pHs (2, 7 and 12) using 0.1% formate buffer, 0.1% ammonium acetate, and 10 mM triethylamine.^35^

##### 4.1.3.4 Log *k*_w_^IAM^

This descriptor required the use of an IAM.PC.DD2 (300 Å, 10 μm, 10 cm × 4.6 mm) column from REGIS. The organic solvent of the eluent, ACN, was modified at various percentages (from 10 to 50%, v/v) and the retention times were measured. Next, the capacity factor (log k′) values were calculated as described elsewhere and the equation represented by the five was obtained. Lastly the extrapolated value at 100% ACN (0% aqueous buffer) or log *k*_w_^IAM^ was obtained. Caffeine, carbamazepine, ketoprofen, theobromine, and toluene were checked daily as gold standards.^39,50,51^

##### 4.1.3.5 ΔLog *k*_w_^IAM^

This polarity descriptor was calculated using the equation Δlog *k*_w_^IAM^ = log *k*_w_^IAM^ – clog *k*_w_^IAM^. Clog *k*_w_^IAM^, defined as the log *k*_w_^IAM^ for neutral compounds with PSA = 0, has been correlated to BRlogD by our group with the equation: clog *k*_w_^IAM^= 0.92*BRlogD – 1.03.^39^

##### 4.1.3.6 Chamelogk

Using the PLRP-S column, we assessed the RT of each PROTAC in the data set at four different mobile phase conditions (50, 60, 70 and 100% MeCN) and calculated the capacity factor at each mobile phase composition. First, we constructed a linear trend using the first three mobile phase conditions (50, 60, and 70% MeCN) that respected a linear behavior and calculated the extrapolated value at 100% MeCN (Ext. log k′100). Chamelogk was then calculated with the following equation: Chamelogk = Exp. log k′100 – Ext. log k′100.^15^

### 4.2 Computational Part

#### 4.2.1 2D Descriptors

The simplified molecular input line-entry system (SMILES) codes of the 11 PROTACs were provided by Boehringer Ingelheim. Marvin was used for drawing, displaying and characterizing chemical structures, substructures and reactions, Marvin version 22.13.0, 2022, ChemAxon (http://www.chemaxon.com).^52^ Their 2D molecular properties were calculated. Their molecular weight (MW), number of carbon atoms (nC), topological polar surface area (TPSA), number of hydrogen bond acceptors (HBA) and donors (HBD), and Kier flexibility index (Φ or PHI) were calculated using AlvaDesc (https://www.alvascience.com/alvadesc).^53,54^ Notably, the HBDs were calculated by the software by adding up the hydrogen atoms bonded to each nitrogen and oxygen without negative charge in the molecule. In addition, the HBAs were calculated as the sum of all nitrogen, oxygen, and fluorine atoms. Moreover, the number of rotatable bonds (NRotB), the number of aromatic rings (NAR) and the logarithms of the octanol/water partition coefficient (cLogP) were calculated using DataWarrior (v06.0.0).^55^ The p*K*_a_ of the compounds at pH 7.0 was calculated using Marvin version 22.13.0, 2022, ChemAxon (http://www.chemaxon.com).^52^

#### 4.2.2 3D Descriptors (CS and SMD)

The starting 3D geometries were obtained using CORINA demo (www.mnam.com/online-demos/corina_demo). The SMILES codes were submitted after verifying chirality and bond orders. The resulting 3D structures were also checked for their stereochemistry, bond order, protonation (if applicable), and correct bond distances and angles. Planarity in aromatic groups was checked. The resulting 3D structures were submitted to different conformational sampling strategies.

First, a mixed torsional/low-mode sampling (MCMM/LMOD) algorithm, which uses the OPLS_2005 force field, followed by an implicit solvent minimization in water (polar) and chloroform (nonpolar), using a generalized Born (GB)/surface areas (SA) solvation model. For this purpose, the Maestro suite was used (Schrödinger, release 2021-3; Maestro, version 12.3, Maestro LLC, New York, NY, 2021, and Schrödinger release 2021-3, Macromodel, LLC, New York, NY, 2021). Additional details can be found in the referenced literature.^19–21^

Second, molecular dynamics was used, in particular steered molecular dynamics.^20,21^ The online input generator CHARMM-GUI (www.charmm-gui.org) was used to prepare the equilibration inputs in water and toluene. First the 3D structures were prepared with the “ligand reader & modeler function”. Next, the solvent box was built using the “solution builder” function, which allowed to create a periodic 50–70 Å water box. The periodic toluene box (nonpolar solvent) was made with the “multicomponent assembler” toolkit (toluene density at RT = 870 g/L). Lastly, both the water and toluene input simulations were prepared for runs under NPT conditions and 300 K. The inputs were downloaded and transferred to a Linux system (130 GiB system memory, Samsung SSD 980 PRO 1TB, 32 cores, AMD Ryzen 9 5950X 16-Core Processor, NVIDIA Corporation GA106 [RTX A2000] GPU) in which NAMD2 CUDA-accelerated software (www.ks.uiuc.edu/Research/namd/)^56^ was used to perform the equilibrations for 0.25 ns. Subsequently, the production runs were performed starting from open and close conformations for 20 and 150 ns, in order to guarantee reproducibility. Steered conditions were applied to the PROTAC by adding to the production script the following parameters: SMD = on, SMDk = 7.0 kcal/mol/Å, SMDvel = 2e^–05^ Å/ts and SMDdir = 0.0, 1.0, 0.0. Moreover, to apply steered conditions only to the PROTAC structure, the occupancy factor of its atoms (pdb file) was changed to 1. The SMD productions were launched and successfully handled with VMD (http://www.ks.uiuc.edu/Research/vmd/).^57^ The solvent (water or toluene) was removed, and the clean trajectories were obtained after checking that SMD did not break any bond or induce abnormal atomic clashes.

For the obtained frames (CS and SMD), several 3D descriptors were calculated with VEGA ZZ:^58^ polarity in 3D (3D PSA 0 Å), and 3D PSA 1.4 Å using a probe radius of 1.4 Å, and *R*_gyr_, as an indicator of sphericity. IMHBs were calculated with the default method in USCF Chimera 1.15 (https://www.rbvi.ucsf.edu/chimera/)^59^ as reported elsewhere.^21^

#### 4.2.3 Graphical Analysis

Statistical relationships between the experimental data and 2D and 3D descriptors were explored with DataWarrior^55^ and R Studio (version 2023.06.2, https://posit.co). Graphical plotting was also performed with both software.

### 4.3 Compounds

The detailed synthesis of compounds A5–1, A5–2, A5–3, A3–1, B-1, B-2, B-4 was disclosed in an earlier publication.^12^

The synthesis of compounds A5–4, A3–2, A3–3, B-3 is described elsewhere.^60^ Analytical data (HRMS, NMR) can be found in the SI.

All compounds are >95% pure by HPLC. HPLC traces are included in the SI.

## Supplementary Material





## Notes

Molecular formula strings, calculated 2D molecular descriptors and complementary experimental data; Log k′80 PLRP-S of the 11 PROTACs at acid, neutral and basic pH; correlation between passive cellular permeability and F%; SMD-derived property space analysis, longer SMD and SMD starting from folded conformations, polarity and sphericity median values in toluene and water, individually compared by PROTAC series and colored by their oral F%; analytical data and HPLC traces (PDF)
Class, series, public name, and SMILES (CSV)

## Abbreviations Used

bRo5: beyond rule of 5
CRBN: cereblon
CS: conformational sampling
ER: efflux ratio
ETR: EPSA-to-TPSA ratio
IMHB: intramolecular hydrogen bond
MMS: matched molecular series
MD: molecular dynamics
POI: protein of interest
PROTAC: proteolysis-targeting chimera
*R*_gyr_: radius of gyration
PSA: polar surface area
SMD: steered molecular dynamics
TPSA: topological polar surface area
VHL: von Hippel–Lindau
