# Supplementary material for: Linker Methylation as a Strategy to Enhance PROTAC Oral Bioavailability: Insights from Molecular Properties and Conformational Analysis
Source: J Med Chem. 2025 Aug 1;68(15):16666–77. doi: 10.1021/acs.jmedchem.5c01497 (PMC12362582; doi:10.1021/acs.jmedchem.5c01497)

Supporting Material

Linker Methylation as a Strategy to Enhance PROTAC Oral Bioavailability: Insights from Molecular Properties and Conformational Analysis

Diego Garcia Jimenez[1], Giuseppe Ermondi[1], Zuzana Jandova[2], Maura Vallaro[1], Giulia Caron[1]*, Heribert Arnhof[2]*

[1] Molecular Biotechnology and Health Sciences Dept., Università di Torino, Piazza Nizza 44bis, 10126, Torino, Italy.

Email: giulia.caron@unito.it, phone number +39 0116706371

[2] Boehringer Ingelheim RCV GmbH Co KG, Dr. Boehringer-Gasse 5-11, 1121 Vienna, Austria.

E-Mail: heribert.arnhof@boehringer-ingelheim.com, phone number +43 1 80105 2515

**Reference authors*

List of contents

Table S1. a Calculated 2D molecular descriptors for the 11-compound dataset. **b** Predicted pK_a values

Table S2. Complementary experimental data for the PROTAC dataset (ER in the presence of a Pgp inhibitor and hepatic clearance)

Figure S1. Log k'80 PLRP-S of the 11 PROTACs at acid, neutral and basic pH faceted by series name (A5, A3 and B).

Figure S2. EPSA vs Δ log k_W^{IAM} relationship.

Figure S3. Correlation between passive cellular permeability and F%.

Figure S4. bRo5 molecules: F% explained by physchem descriptors.

Figure S5. SMD-derived property space analysis.

Figure S6. 150ns SMD simulation representation of Series A5

Figure S7. 20ns SMD simulation representation of Series A5 starting from folded input conformations

Figure S8. Polarity and sphericity median values in toluene and water, individually compared by PROTAC series and colored by their oral F%.

Figure S9. Polarity and sphericity median values in toluene and water, individually compared by PROTAC series and colored by their efflux pump ratio.

Analytical data (HRMS and NMR)

HPLC traces

Table S1| a Calculated 2D molecular descriptors for the 11-compound dataset. **b** Predicted pK_a values

Molecule Name	Published name	Series	MW	nC	PHI	HBD	HBA	TPSA	cLogP	NAR	RtB
**A5-1**	**C11**	**A5**	1050.3	55	14.7	3	14	182.4	9.5	4	16
**A5-2**	**ACBI2**		1064.3	56	15.0	3	14	182.4	9.7	4	16
**A5-3**	**C29**		1064.3	56	15.0	3	14	182.4	9.7	4	16
**A5-4**			1064.3	56	15.0	3	14	182.4	9.7	4	16
**A3-1**	**C6**	**A3**	1022.2	53	13.7	3	14	182.4	8.6	4	14
**A3-2**			1036.2	54	14.0	3	14	182.4	8.8	4	14
**A3-3**			1036.2	54	14.0	3	14	182.4	8.8	4	14
**B-1**	**C24**	**B**	1038.2	53	14.2	3	15	191.6	7.6	4	15
**B-2**	**C9**		1052.2	54	14.4	3	15	191.6	8.0	4	15
**B-3**			1052.2	54	14.4	3	15	191.6	8.0	4	15
**B-4**	**C28**		1066.3	55	14.4	3	15	191.6	8.4	4	15

Molecule Name	Published name	Series	Calculated pK_a (Acid)	Calculated pK_a (Basic)
**A5-1**	**C11**	**A5**	11.28	9.71, 2.99, 2.39
**A5-2**	**ACBI2**		11.28	9.89, 2.99, 2.39
**A5-3**	**C29**		11.28	9.89, 2.99, 2.39
**A5-4**			11.29	9.89, 2.99, 2.39
**A3-1**	**C6**	**A3**	11.27	9.33, 2.99, 2.36
**A3-2**			11.22	9.64, 2.99, 2.36
**A3-3**			11.22	9.64, 2.99, 2.36
**B-1**	**C24**	**B**	11.16	8.81, 2.99, 2.39
**B-2**	**C9**		11.16	9.12, 2.99, 2.39
**B-3**			11.16	9.12, 2.99, 2.39
**B-4**	**C28**		11.17	9.43, 2.99, 2.39

Table S2. Complementary experimental data for the PROTAC dataset (ER in the presence of a Pgp inhibitor and hepatic clearance)

PROTAC	Series	EPSA	ER (+Pgp inhibitor)	Mouse CL (% Qh)
**A5-1**	**A5**	136	1.6	2.5
**A5-2**		133	1.6	2.3
**A5-3**		133	1.0	3.9
**A5-4**		134	0.9	2.8
**A3-1**	**A3**	133	1.8	7.9
**A3-2**		130	0.9	3.6
**A3-3**		130	0.4	9.3
**B-1**	**B**	128	3.0	8.5
**B-2**		na	1.8	5.4
**B-3**		124	2.2	7.1
**B-4**		127	1.1	6.0

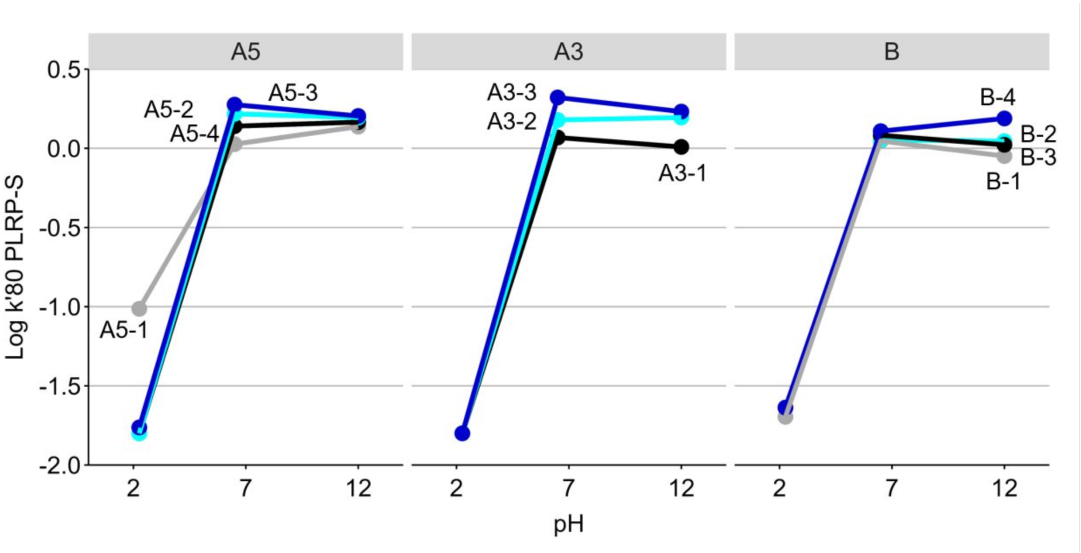

Figure S1. Log k'80 PLRP-S of the 11 PROTACs at acid, neutral and basic pH faceted by series name (A5, A3 and B).

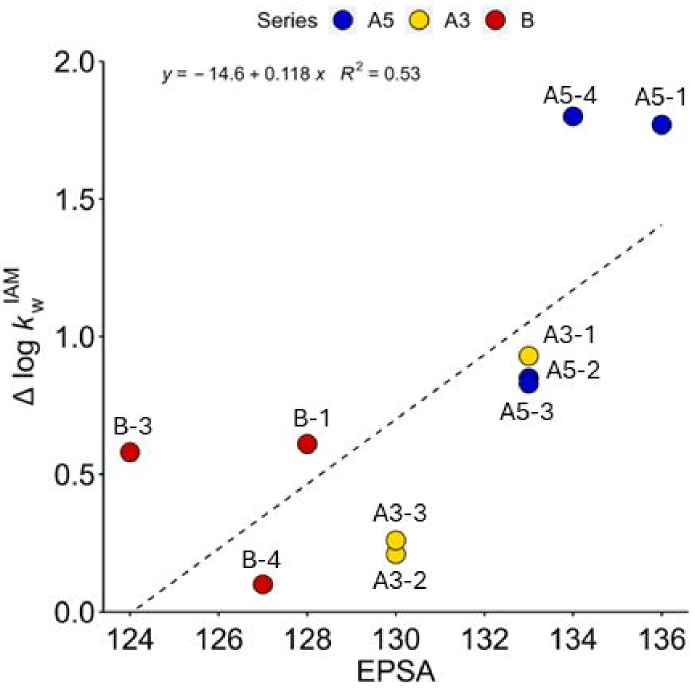

Figure S2. EPSA vs Δ log k_W^{IAM} representation.

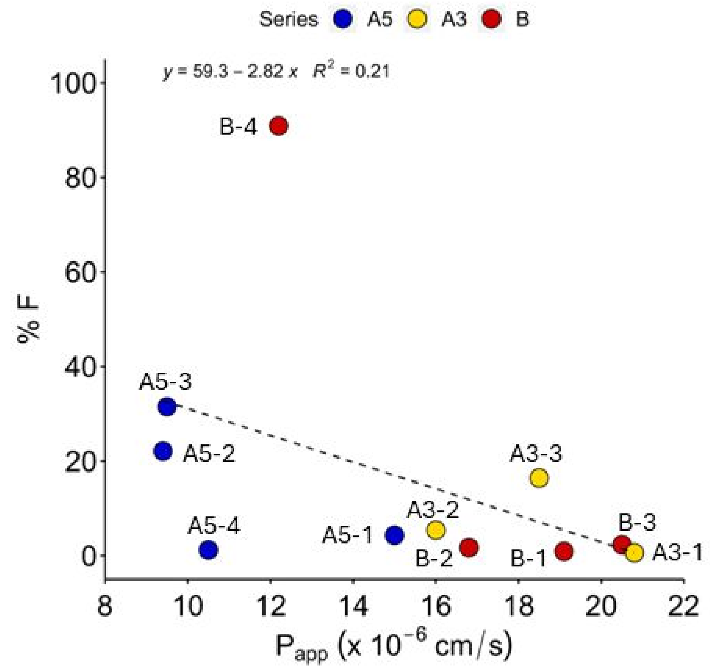

Figure S3. Correlation between passive cellular permeability and F%.

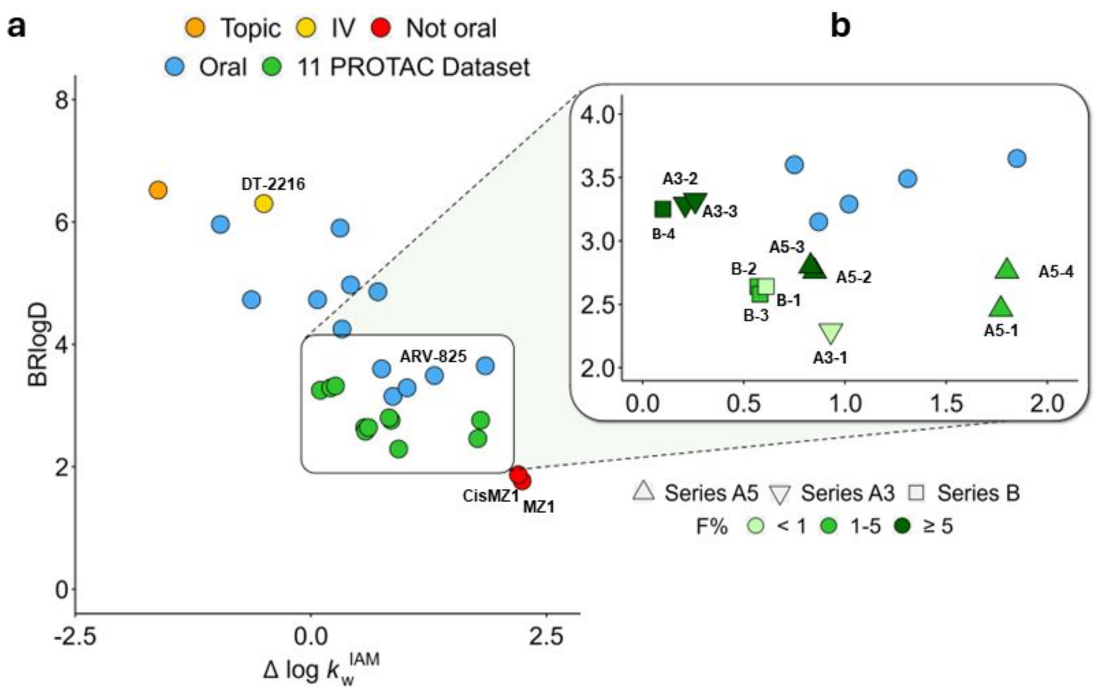

Figure S4. bRo5 molecules: F% explained by physchem descriptors. a BRlogD vs Δ log k_W^{IAM} plot colored by absorption or F% groups. **b** zoomed plot of the 11 PROTAC Dataset.

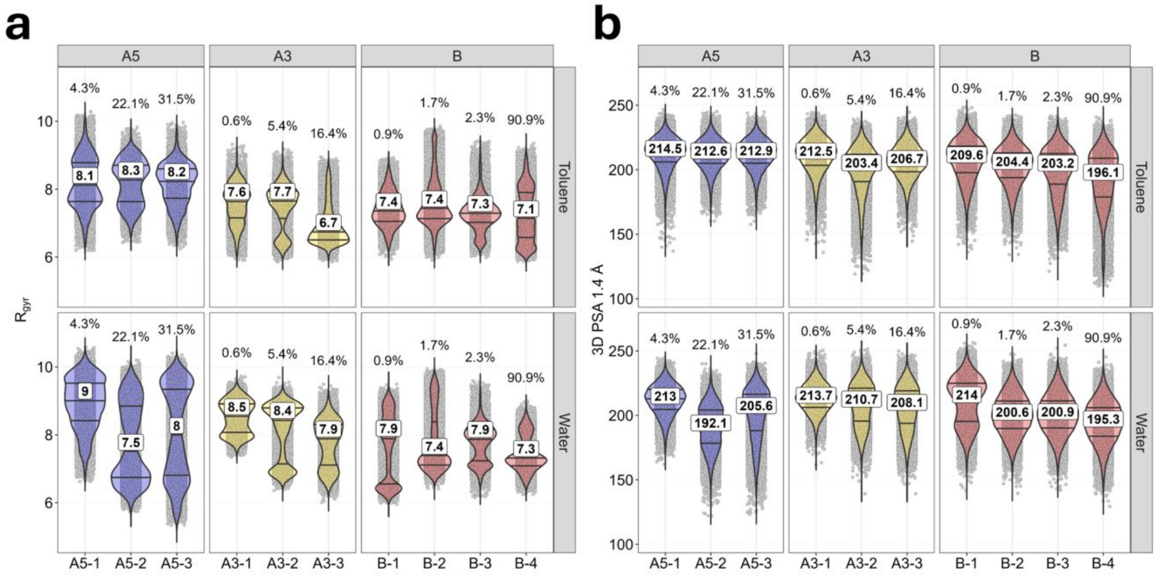

Figure S5. *SMD-derived property space analysis* **a** ***and b*** *Violin plots for all series divided by solvent (R_{gyr} and 3D PSA, respectively)(20 ns). Median values are labeled. This figure confirms that PROTACs with the methyl groups in the linker (A5-2, A5-3, A3-2 and A3-3) in water tend to reach the most folded conformations (lowest R_{gyr}) and the lowest polarity. Furthermore, the influence of the methyl group on conformation is more pronounced in PROTACs with a longer linker.*

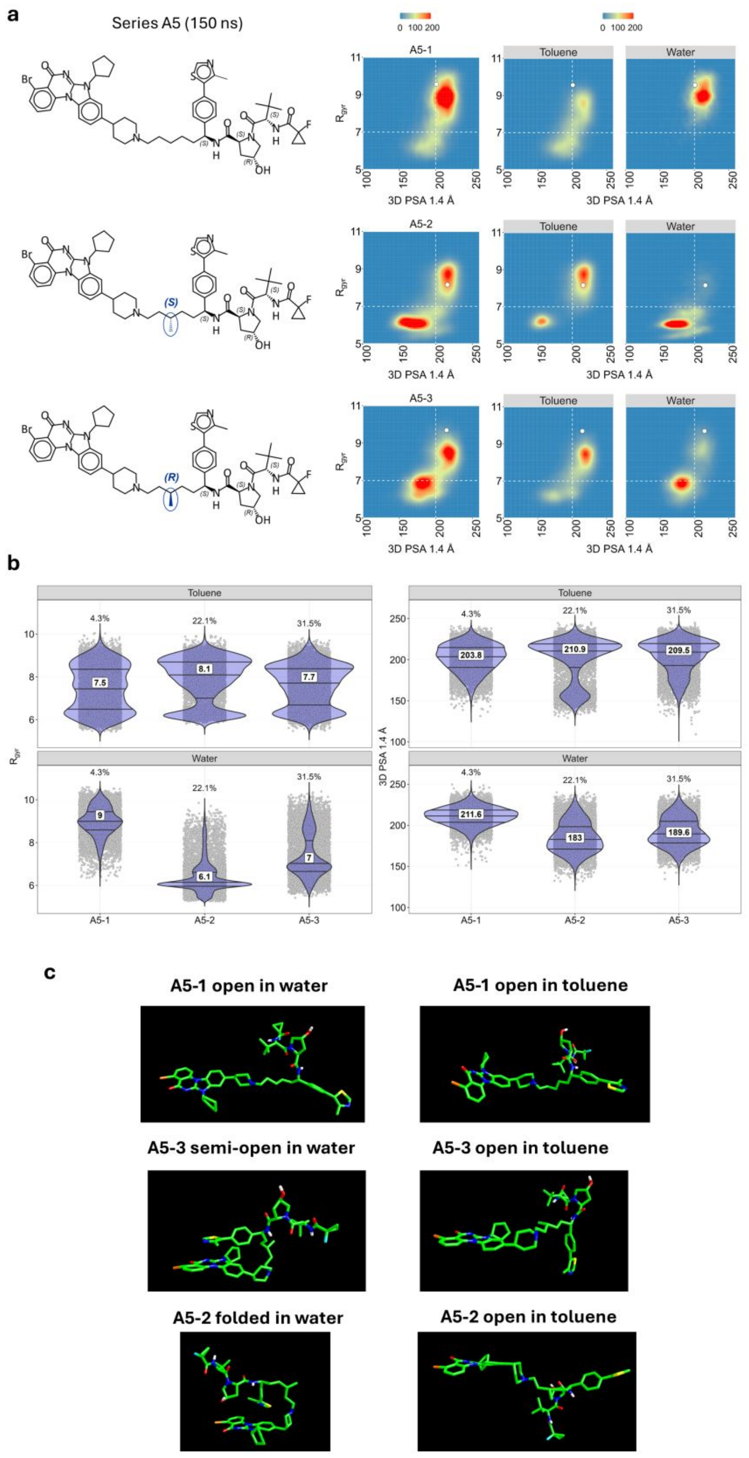

Figure S6. *150ns SMD simulation representation of Series A5.* ***a*** *Conformers are represented as a function of their 3D PSA 1.4 Å and* R_{gyr} *and colored by conformer count. White dots represent the starting conformations of the SMD simulations. Starting conformers are colored in white, respectively.* ***b*** *3D-property distribution, represented by violin plots. Median values are labelled.* ***c*** *conformers representing density maximum in water and toluene: their step numbers are: A5-*1_w*= 8692, A5-*1_t*= 6777, A5-*2_w*= 330, A5-*2_t*= 4991, A5-*3_w*= 9831, C*29_t*= 2481.*

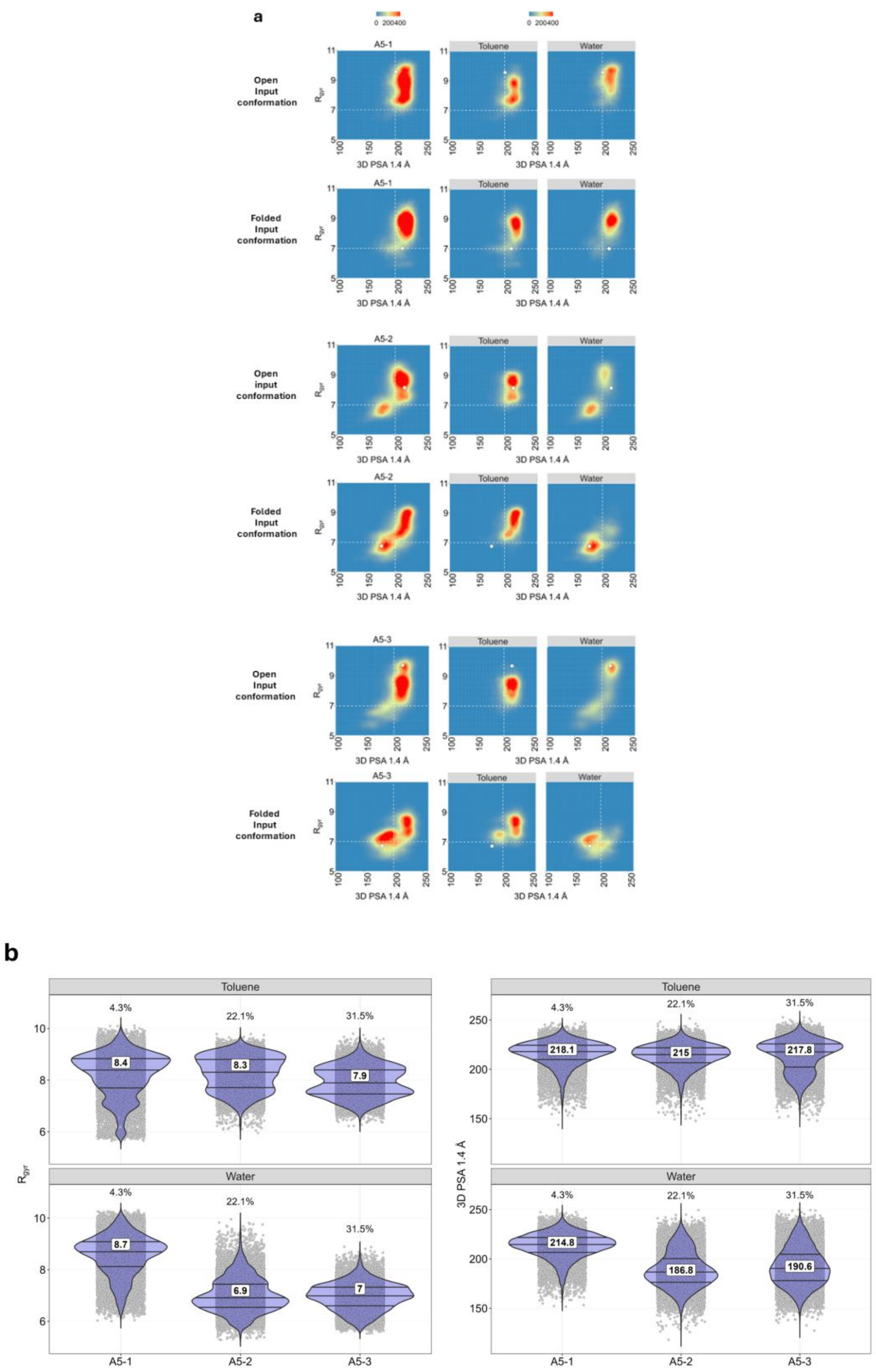

Figure S7. 20ns SMD simulation representation of Series A5 starting from folded input conformations. **a** Conformers are represented as a function of their 3D PSA 1.4 Å and R_{gyr} and colored by conformer count. **b** 3D-property distribution, represented by violin plots. Median values are labelled.

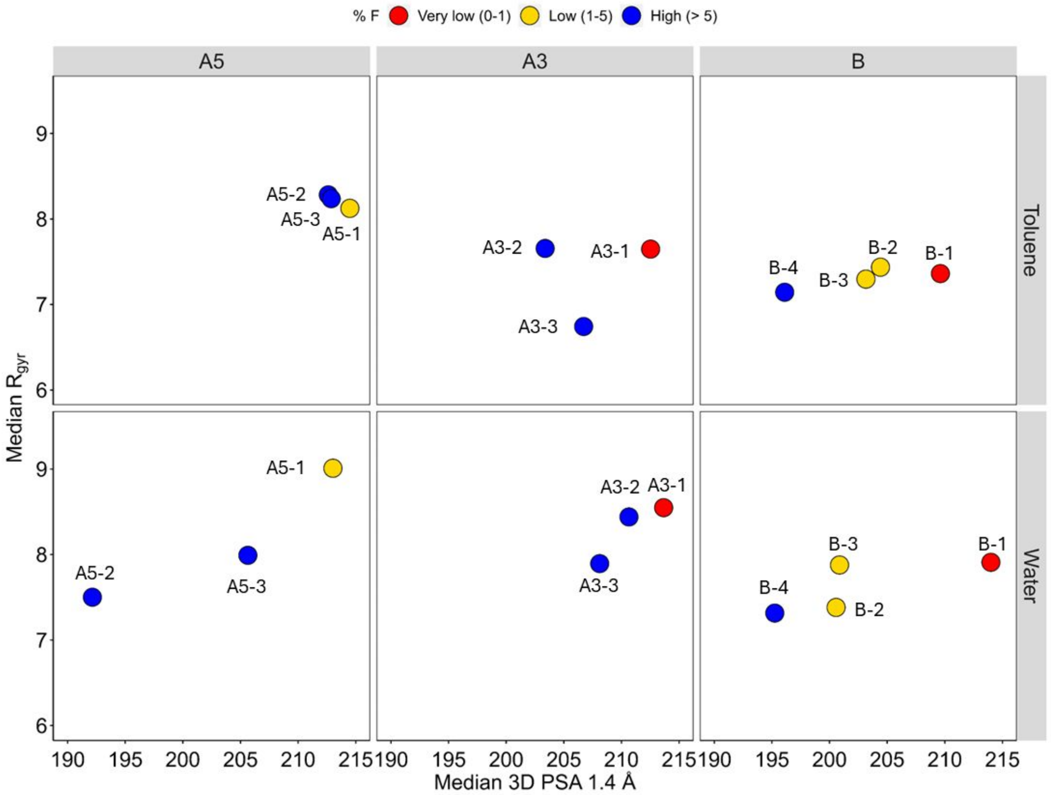

Figure S8. Polarity and sphericity median values in toluene and water, individually compared by PROTAC series and colored by their oral F%.

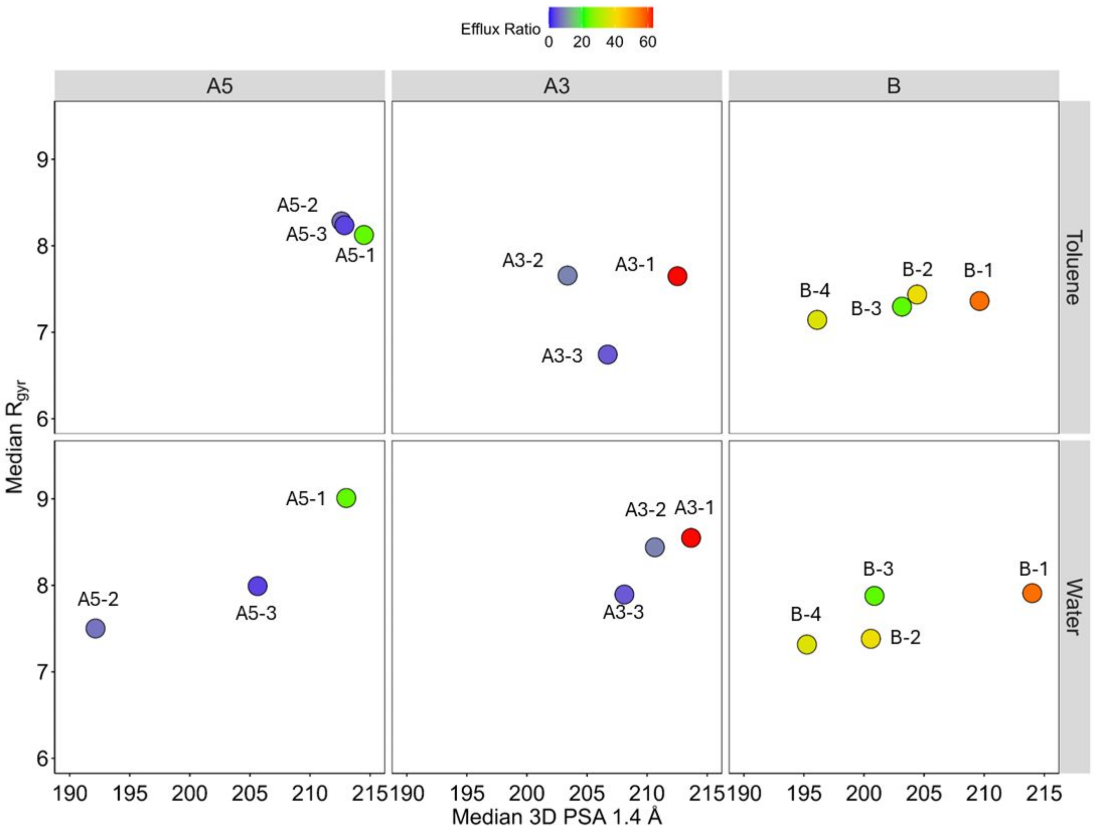

Figure S9. Polarity and sphericity median values in toluene and water, individually compared by PROTAC series and colored by their efflux pump ratio.

Analytical Data

NMR experiments:

NMR experiments were recorded on Bruker Avance 600 MHz spectrometer at 298 K. Samples were dissolved in 600 µL DMSO-d_6 and TMS was added as an internal standard. 1D ^{1}H spectra were acquired with 30° excitation pulses and an interpulse delay of 4.2 s with 64 k datapoints and 20 ppm sweep width. 1D ^{13}C spectra were acquired in modulated mode with broadband composite pulse decoupling (WALTZ16) and an interpulse delay of 3.3 s with 64 k datapoints and a sweep width of 240 ppm. Processing and analysis of 1D spectra was performed with Bruker Topspin 3.0 software. No zero filling was performed, and spectra were manually integrated after automatic baseline correction. ^{13}C DEPT modulated spectra were phased so that C, CH2 are positive, and CH, CH3 are negative. 2D HSQC spectra were recorded on all samples to aid the interpretation of the data and to identify signals hidden underneath solvent peaks. Spectra were acquired with sweep widths obtained by automatic sweep width detection from 1D reference spectra in the direct dimension with 1k datapoints and with 210 ppm and 256 datapoints in the indirect dimension. Spectra were processed with Topspin 3.6 software from Bruker and analyzed with ACDlabs NMR workbook 2023.

In certain cases, it was not possible to differentiate between peaks arising from rotamers and those arising from weak intensities due to either slow relaxing quaternary carbons and/or other causes, which is why the number of peaks in the carbon peak list is not always consistent with the number of carbons in the molecule. The same holds true for the proton intensities where due to overlapping rotamers the integrals are slightly smaller or larger than the expected number of protons.

A5-4

HRMS (ESI+) *m/z*: $[M+H]^+$ calcd for C56H68BrFN8O5S 1063.42736; found 1063.42786

^{1}H NMR (600 MHz, DMSO-d_6) δ: 8.97 (s, 1H), 8.46 (d, J=8.6 Hz, 1H), 8.42 (br d, J=8.3 Hz, 1H), 8.32-8.37 (m, 0.2H rotamer), 8.28 (d, J=8.6 Hz, 1H), 7.79 (d, J=7.7 Hz, 1H), 7.73 (br d, J=9.4 Hz, 0.1H rotamer), 7.67 (t, J=8.1 Hz, 1H), 7.54 (s, 1H), 7.49 (d, J=8.1 Hz, 2H), 7.42 (d, J=8.3 Hz, 2H), 7.39 (br d, J=8.3 Hz, 1H rotamer), 7.27 (br d, J=8.1 Hz, 1H), 7.25 (br dd, J=9.4, 2.4 Hz, 1H), 7.02 (br dd, J=9.0, 2.9 Hz, 1H rotamer), 5.25 (quin, J=8.8 Hz, 1H), 5.17 (br s, 1H), 5.13 (br d, J=2.4 Hz, 0.1H rotamer), 4.72-4.82 (m, 1H), 4.65-4.71 (m, 0.1H rotamer), 4.52-4.60 (m, 2H), 4.48-4.51 (m, 0.2H rotamer), 4.36 (br s, 1H), 3.63-3.70 (m, 1H), 3.55-3.62 (m, 1H), 2.98 (br d, J=11.0 Hz, 2H), 2.64-2.73 (m, 1H), 2.45 (s, 3H), 2.29 (br d, J=7.3 Hz, 4H), 2.10 (br dd, J=12.5, 7.7 Hz, 1H), 1.90-2.04 (m, 7H), 1.73-1.82 (m, 6H), 1.45-1.52 (m, 2H), 1.33-1.40 (m, 2H), 1.14-1.26 (range, 3H), 0.99-1.01 (m, 0.4H rotamer), 0.94-0.96 (m, 0.61H rotamer), 0.91 (s, 8H), 0.89 (br s, 3H), 0.88 (br s, 1H rotamer)

^{13}C NMR (150 MHz, DMSO-d_6) δ: 170.5, 168.2, 167.4 (d, CF=20.3 Hz), 163.5, 150.8, 148.2, 147.1, 143.0, 142.7, 138.2, 132.7, 131.5, 130.6, 129.9, 129.0, 128.0, 126.4, 124.2, 122.2, 120.6, 116.0, 114.8, 113.0, 108.6, 77.5 (d, CF=231.4 Hz), 68.3, 58.2, 56.0, 55.9, 55.6, 53.5, 53.1, 51.7, 41.4, 37.4, 35.4, 32.9, 32.8, 32.8, 32.5, 29.7, 27.4, 25.8, 25.6, 23.9, 19.3, 15.3, 12.4 (d, CF=10.2 Hz), 12.1 (d, CF=10.2 Hz)

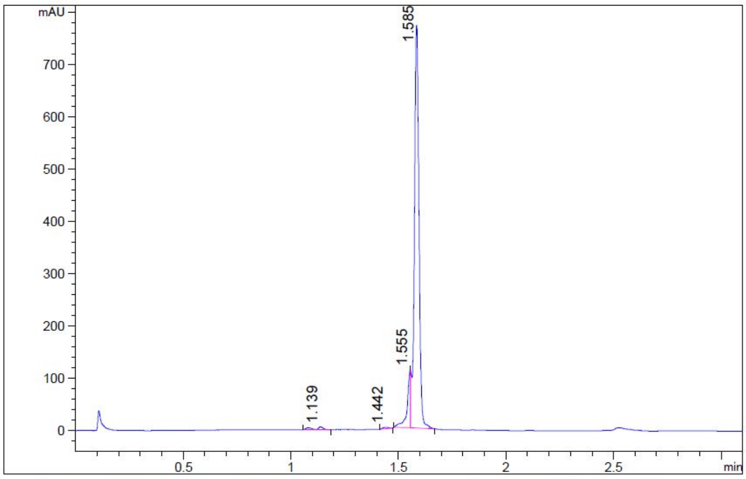


```
RT:   1.14 min                    Area %:     1.1 %
RT:   1.44 min                    Area %:     0.4 %
RT:   1.56 min                    Area %:     8.8 %
RT:   1.59 min                    Area %:    89.6 %
```

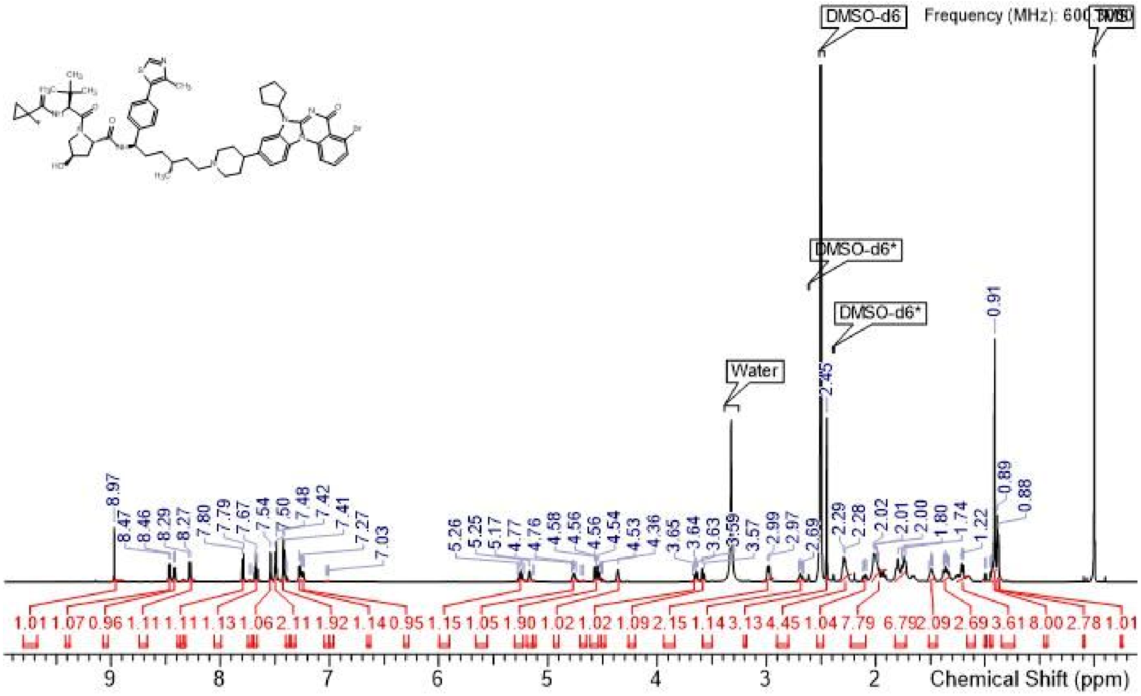
DMSO-d6
Frequency (MHz): 600
TMS
DMSO-d6*
DMSO-d6*
Water
Chemical Shift (ppm)

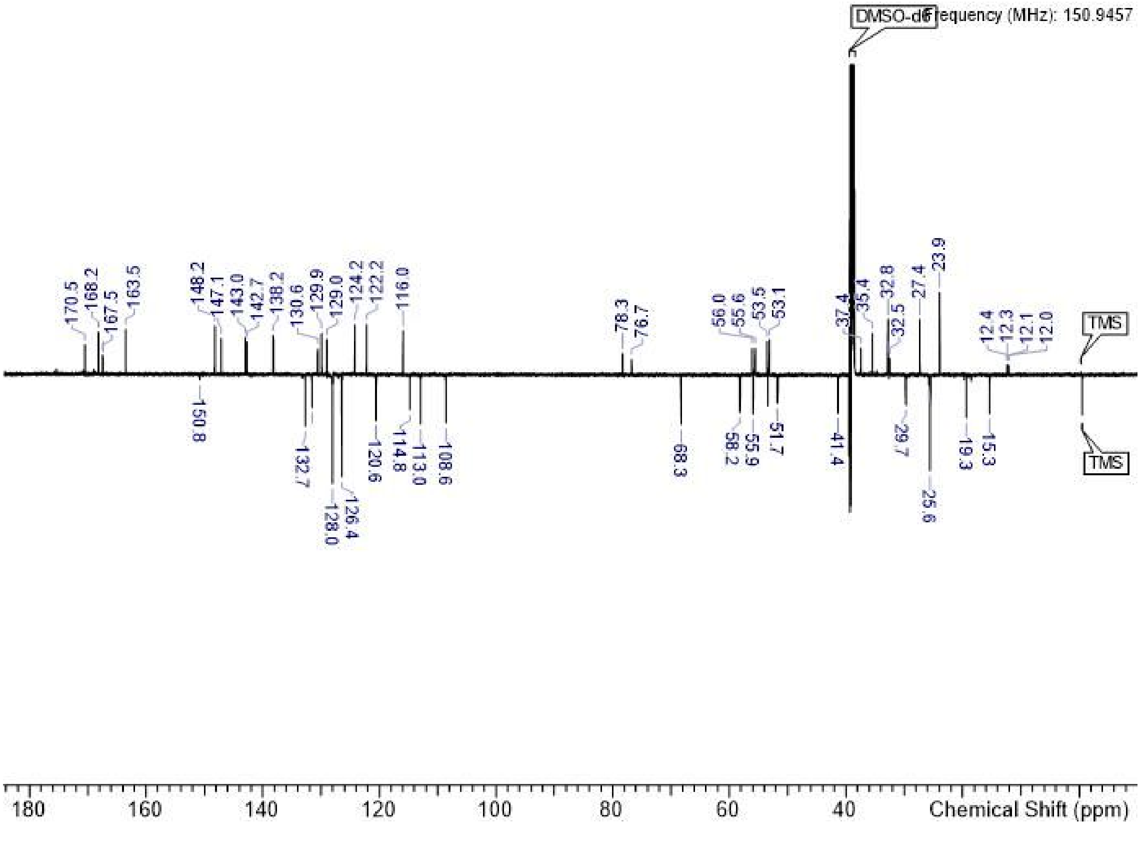
DMSO-d6
Frequency (MHz): 150.9457
TMS
TMS
Chemical Shift (ppm)

A3-2

HRMS (ESI+) *m/z*: $[M+H]^+$ calcd for C54H64BrFN8O5S 1035.39606; found 1035.39648

^{1}H NMR (600 MHz, DMSO-d_6) δ: 8.97 (s, 1H), 8.77 (br d, J=7.3 Hz, 0.1H rotamer), 8.49 (br d, J=8.1 Hz, 1H), 8.47 (d, J=8.6 Hz, 1H), 8.30 (d, J=8.6 Hz, 1H), 7.80 (d, J=7.5 Hz, 1H), 7.68 (t, J=8.2 Hz, 1H), 7.50 (s, 1H), 7.43-7.46 (m, 2H), 7.38-7.41 (m, 2H), 7.36 (br d, J=8.3 Hz, 0.2H rotamer), 7.22-7.30 (m, 2H), 6.95 (dd, J=8.7, 2.7 Hz, 0.1H rotamer), 5.26 (quin, J=8.8 Hz, 1H), 5.13 (d, J=3.7 Hz, 1H), 4.95-5.06 (m, 1H), 4.71-4.81 (m, 0.1H rotamer), 4.67 (t, J=7.3 Hz, 1H), 4.57 (d, J=9.2 Hz, 1H), 4.49 (t, J=8.2 Hz, 2H), 4.45 (br d, J=9.0 Hz, 0.2H rotamer), 4.27 (br s, 1H), 3.52-3.61 (m, 2H), 2.96 (br s, 1H), 2.72-2.83 (m, 1H), 2.64-2.70 (m, 1H), 2.43-2.46 (m, 3H), 2.26 (br dd, J=11.6, 8.7 Hz, 3H), 2.13 (br d, J=10.8 Hz, 1H), 1.96-2.08 (m, 6H), 1.91 (br d, J=4.4 Hz, 1H), 1.64-1.85 (m, 8H), 1.51-1.61 (m, 1H), 1.30-1.41 (m, 2H), 1.21 (br dd, J=8.2, 2.8 Hz, 2H), 0.98 (br s, 1H rotamer), 0.94-0.97 (m, 9H)

^{13}C NMR (150 MHz, DMSO-d_6) δ: 170.9, 169.1, 168.5 (d, CF=20.3 Hz), 164.6, 152.0, 149.3, 148.2, 144.8, 144.0, 139.3, 133.8, 132.6, 131.6, 130.8, 130.2, 129.3, 127.3, 125.3, 123.3, 121.5, 117.0, 115.8, 114.1, 109.6, 78.6 (d, CF=232.7 Hz), 69.2, 65.7, 59.1, 57.1, 57.0, 54.4, 54.4, 51.2, 43.0, 42.3, 38.2, 36.5, 28.4, 27.2, 26.7, 25.1, 19.8, 16.4, 13.4 (d, CF=10.2 Hz)13.2 (d, CF=10.2 Hz)

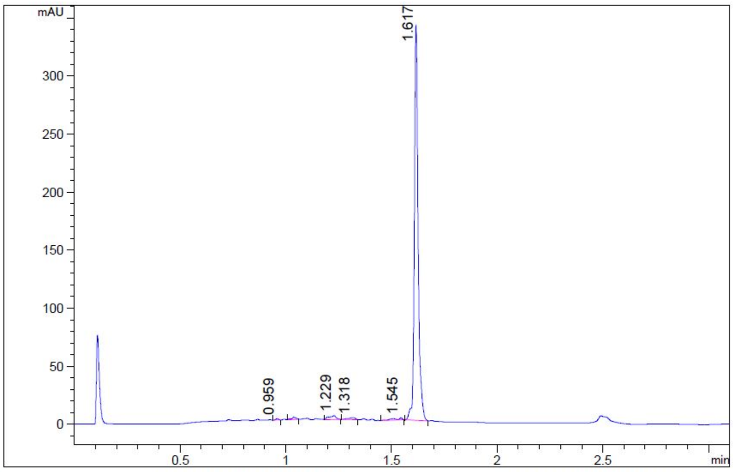

RT: 0.96 min Area %: 0.2 %
RT: 1.04 min Area %: 0.7 %
RT: 1.23 min Area %: 1.7 %
RT: 1.32 min Area %: 0.8 %
RT: 1.55 min Area %: 1.0 %
RT: 1.62 min Area %: 95.6 %

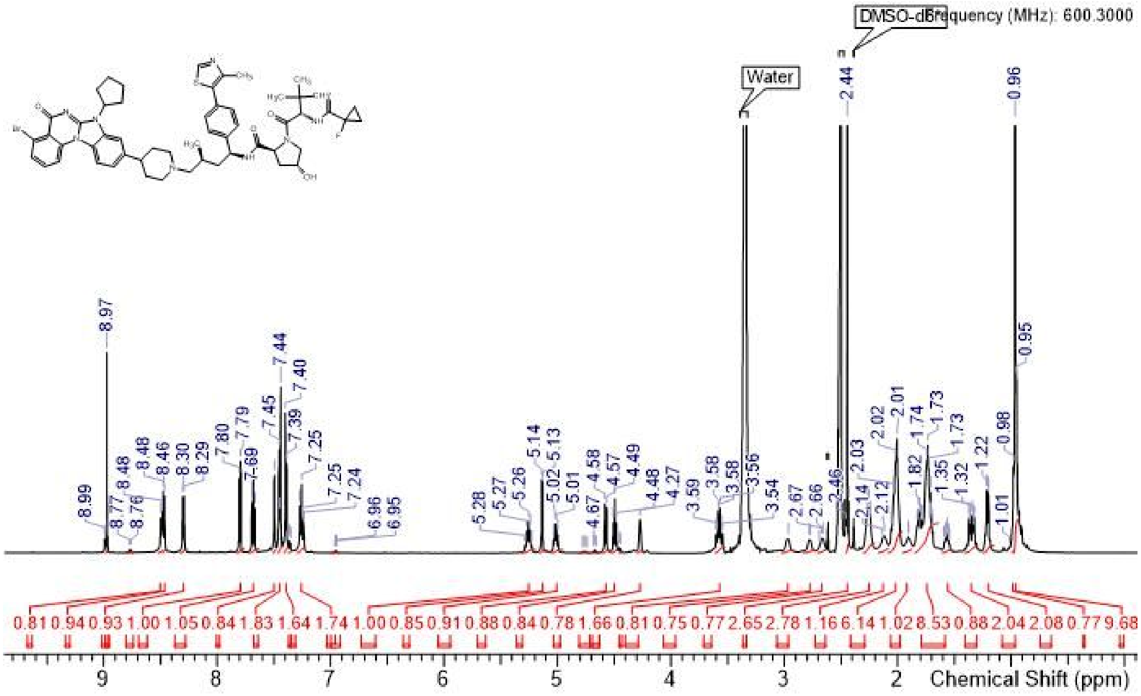

DMSO-d6
Frequency (MHz): 600.3000
Water
Chemical Shift (ppm)

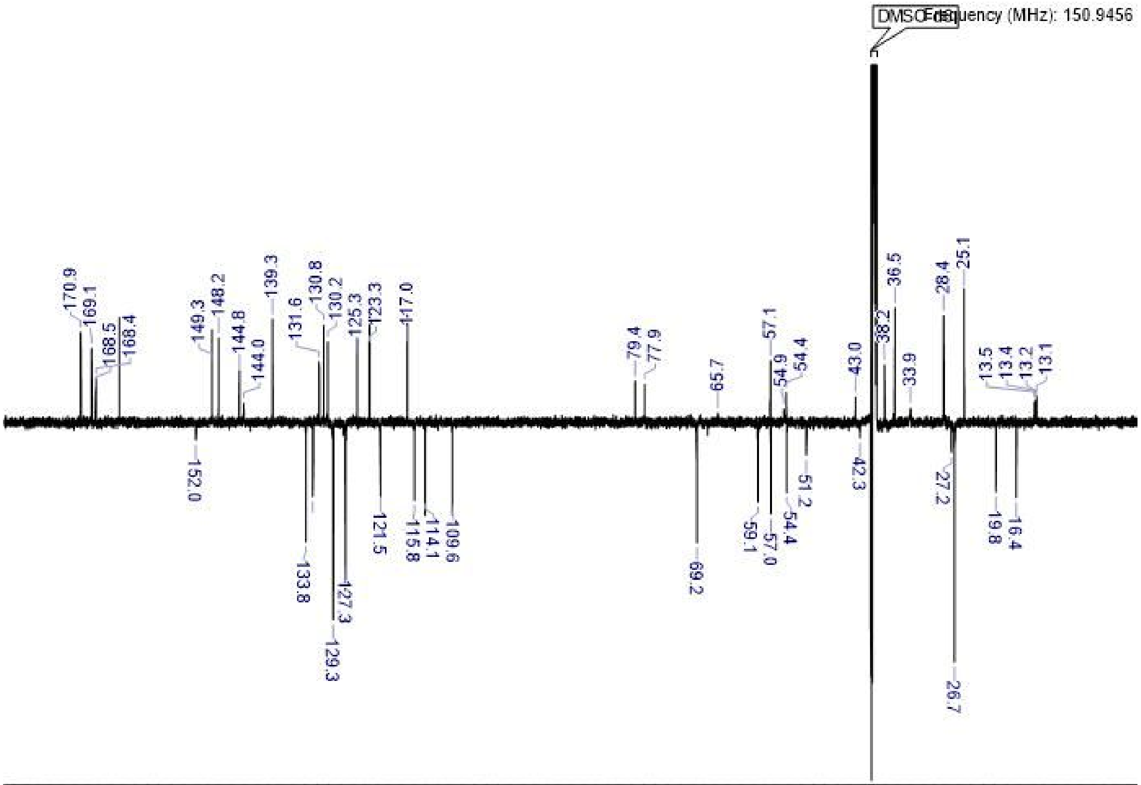

DMSO-d6
Frequency (MHz): 150.9456
Chemical Shift (ppm)

A3-3

HRMS (ESI+) *m/z*: $[M+H]^+$ calcd for C54H64BrFN8O5S 1035.39606; found 1035.39636

^{1}H NMR (600 MHz, DMSO-d_6) δ: 8.99 (s, 1H), 8.97 (s, 0.1H rotamer), 8.64 (br d, J=7.7 Hz, 1H), 8.53 (br d, J=9.0 Hz, 1H), 8.47 (d, J=8.6 Hz, 1H), 8.30 (d, J=8.6 Hz, 1H), 7.80 (d, J=7.7 Hz, 1H), 7.68 (t, J=8.2 Hz, 1H), 7.51 (s, 1H), 7.44-7.48 (m, 2H), 7.39-7.43 (m, 2H), 7.35 (br d, J=8.3 Hz, 0.1H rotamer), 7.28 (d, J=8.6 Hz, 1H), 7.16 (dd, J=9.2, 2.8 Hz, 1H), 5.27 (quin, J=8.8 Hz, 1H), 5.12-5.16 (m, 1H), 5.05-5.12 (m, 1H), 4.57 (d, J=9.2 Hz, 1H), 4.51 (t, J=8.2 Hz, 1H), 4.32 (br s, 1H), 4.21 (br s, 0.1H rotamer), 3.60-3.66 (m, 1H), 3.54-3.60 (m, 1H), 3.24 (br d, J=9.9 Hz, 1H), 3.04 (br d, J=8.3 Hz, 1H), 2.97 (br d, J=2.6 Hz, 0.1H rotamer), 2.71 (br t, J=10.6 Hz, 1H), 2.47 (s, 3H), 2.22-2.33 (m, 2H), 2.10-2.22 (m, 3H), 1.97-2.09 (range, 6H), 1.87-1.96 (range, 1H), 1.67-1.86 (m, 8H), 1.26-1.36 (m, 3H), 1.14-1.24 (m, 2H), 0.98 (s, 8H), 0.95 (br d, J=5.7 Hz, 3H)

^{13}C NMR (150 MHz, DMSO-d_6) δ: 170.2, 168.0, 167.3 (d, CF=20.3 Hz), 163.5, 150.9, 148.2, 147.2, 143.9, 143.0, 138.2, 132.7, 131.5, 130.5, 129.8, 129.1, 128.3, 126.1, 124.2, 122.2, 120.6, 116.0, 114.8, 113.0, 108.4, 77.7 (d, CF=232.7 Hz), 68.3, 58.2, 56.1, 55.9, 54.2, 53.3, 52.9, 48.6, 42.1, 41.4, 37.1, 35.6, 27.4, 25.9, 25.5, 24.0, 24.0, 17.0, 15.4, 12.3 (d, CF=10.2 Hz), 12.1 (d, CF=10.2 Hz)

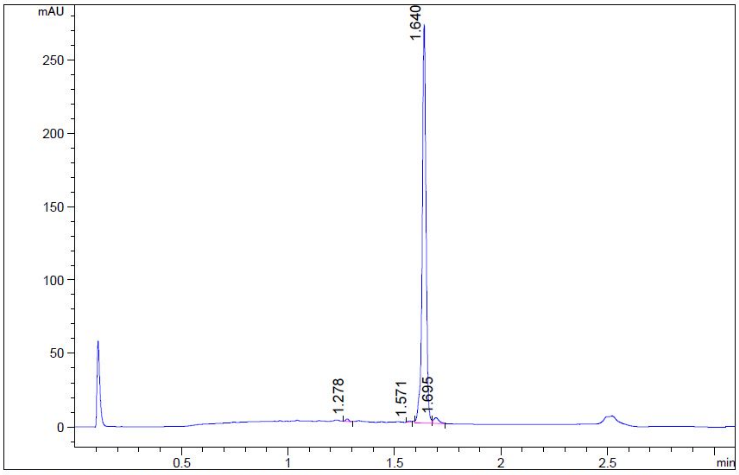

RT: 1.28 min	Area %: 0.5 %
RT: 1.57 min	Area %: 0.2 %
RT: 1.64 min	Area %: 97.4 %
RT: 1.70 min	Area %: 1.9 %

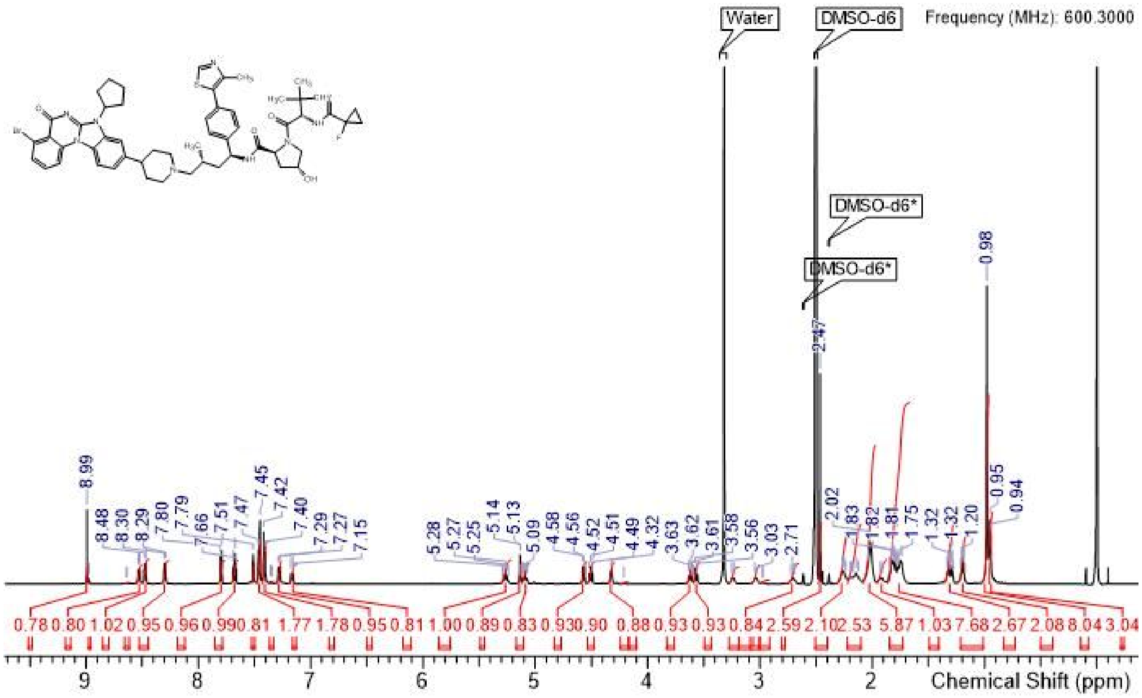
Water
DMSO-d6
Frequency (MHz): 600.3000
DMSO-d6*
DMSO-d6*
0.98
2.47
8.99
8.48
8.30
8.29
7.80
7.79
7.66
7.51
7.47
7.45
7.42
7.40
7.29
7.27
7.15
5.28
5.27
5.25
5.14
5.13
5.09
4.58
4.56
4.52
4.51
4.49
4.32
3.63
3.62
3.61
3.58
3.56
3.03
2.71
2.02
1.83
1.82
1.81
1.75
1.32
1.32
1.20
0.95
0.94
0.78 0.80 1.02 0.95 0.96 0.99 0.81 1.77 1.78 0.95 0.81 1.00 0.89 0.83 0.93 0.90 0.88 0.93 0.93 0.84 2.59 2.10 2.53 5.87 1.03 7.68 2.67 2.08 8.04 3.04
9
8
7
6
5
4
3
2
Chemical Shift (ppm)

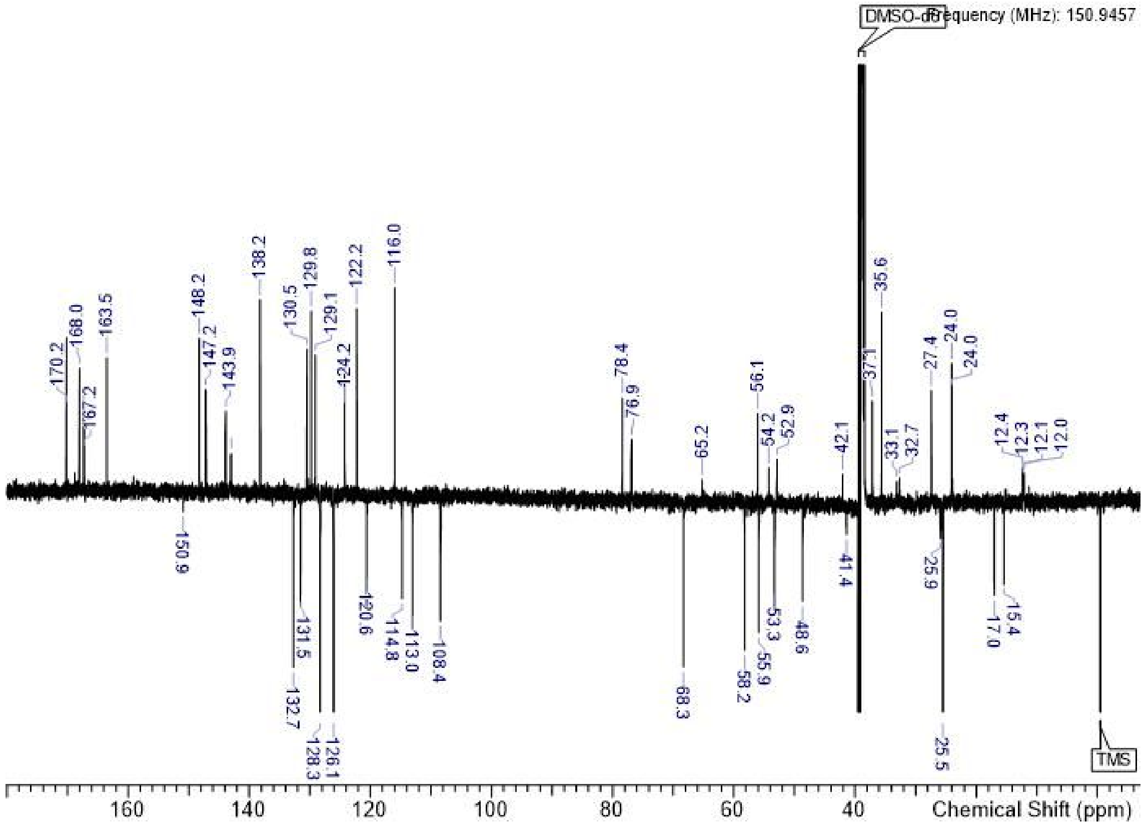
DMSO-d6
Frequency (MHz): 150.9457
170.2
168.0
167.2
163.5
148.2
147.2
143.9
138.2
130.5
129.8
129.1
124.2
122.2
116.0
78.4
76.9
65.2
56.1
54.2
52.9
42.1
37.1
35.6
33.1
32.7
27.4
24.0
24.0
12.4
12.3
12.1
12.0
150.9
131.5
132.7
128.3
126.1
120.6
114.8
113.0
108.4
68.3
58.2
55.9
53.3
48.6
41.4
25.9
25.5
17.0
15.4
TMS
160
140
120
100
80
60
40
Chemical Shift (ppm)

B-3

HRMS (ESI+) ***m/z*****:** $[M+H]^+$ calcd for C54H64BrFN8O6S 1051.39097; found 1051.39185

^{1}H NMR (600 MHz, DMSO-d_6) δ: 8.96 (s, 1H), 8.94 (s, 0.1H rotamer), 8.72 (d, J=8.1 Hz, 0.1H rotamer), 8.46 (d, J=8.8 Hz, 1H), 8.44 (d, J=8.3 Hz, 1H), 8.26 (d, J=8.6 Hz, 1H), 7.80 (d, J=7.7 Hz, 1H), 7.68 (t, J=8.2 Hz, 1H), 7.50 (d, J=0.9 Hz, 1H), 7.42-7.47 (m, 4H), 7.24-7.27 (m, 1H), 7.22-7.24 (m, 1H), 6.98 (dd, J=8.8, 2.9 Hz, 0.1H rotamer), 5.24 (quin, J=8.8 Hz, 1H), 5.15 (d, J=3.7 Hz, 1H), 5.01 (dt, J=8.1, 6.0 Hz, 1H), 4.94-4.98 (m, 0.1H rotamer), 4.78 (t, J=7.6 Hz, 0.1H rotamer), 4.59 (d, J=9.4 Hz, 1H), 4.55 (t, J=8.2 Hz, 1H), 4.39-4.43 (m, 0.1H rotamer), 4.29 (br s, 1H), 4.24-4.27 (m, 0.1H rotamer), 3.80-3.86 (m, 0.2H rotamer), 3.68-3.74 (m, 2H), 3.64-3.68 (m, 1H), 3.59-3.64 (m, 1H), 3.52-3.59 (m, 1H), 3.48 (br d, J=12.3 Hz, 0.1H rotamer), 3.38-3.41 (m, 0.1H rotamer), 2.94 (br dd, J=19.0, 11.1 Hz, 2H), 2.86 (br d, J=10.6 Hz, 0.1H rotamer), 2.41-2.46 (m, 4H), 2.26 (br d, J=5.7 Hz, 3H), 1.94-2.14 (m, 7H), 1.58-1.86 (m, 7H), 1.28-1.42 (m, 2H), 1.16-1.26 (m, 2H), 1.03-1.14 (m, 3H), 0.90-1.03 (m, 9H)

^{13}C NMR (150 MHz, DMSO-d_6) δ: 170.3, 170.1, 168.8, 168.2, 167.4 (d, CF=20.3 Hz), 166.8, 166.6, 163.5, 150.9, 148.2, 147.2, 142.9, 140.3, 138.2, 132.7, 131.5, 130.5, 129.9, 129.4, 128.0, 127.9, 127.0, 126.9, 124.2, 122.2, 120.5, 116.0, 114.7, 112.9, 108.5, 77.5 (d, CF=232.7 Hz), 72.6, 72.3, 70.3, 68.2, 66.3, 63.1, 58.1, 56.0, 55.9, 54.1, 53.6, 53.3, 51.7, 41.1, 37.1, 35.5, 32.8, 32.8, 27.4, 25.6, 24.0, 18.1, 15.4, 12.4 (d, CF=10.2 Hz), 12.1 (d, CF=10.2 Hz)

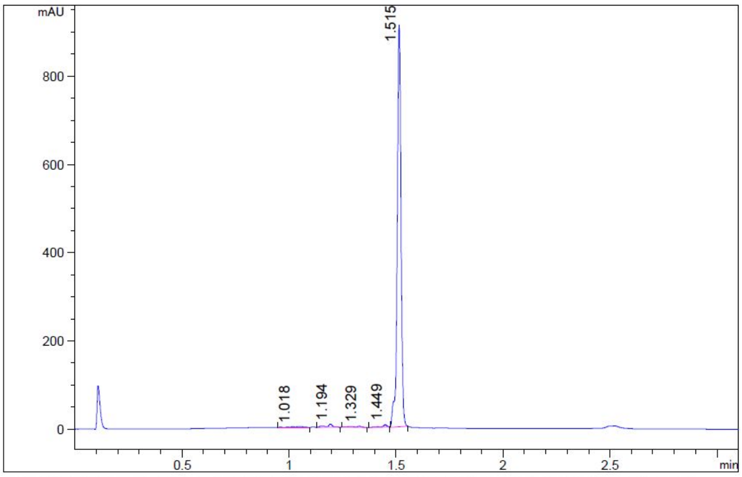

RT:	1.02 min	Area %:	0.5 %
RT:	1.19 min	Area %:	1.1 %
RT:	1.33 min	Area %:	0.6 %
RT:	1.45 min	Area %:	0.7 %
RT:	1.51 min	Area %:	97.1 %

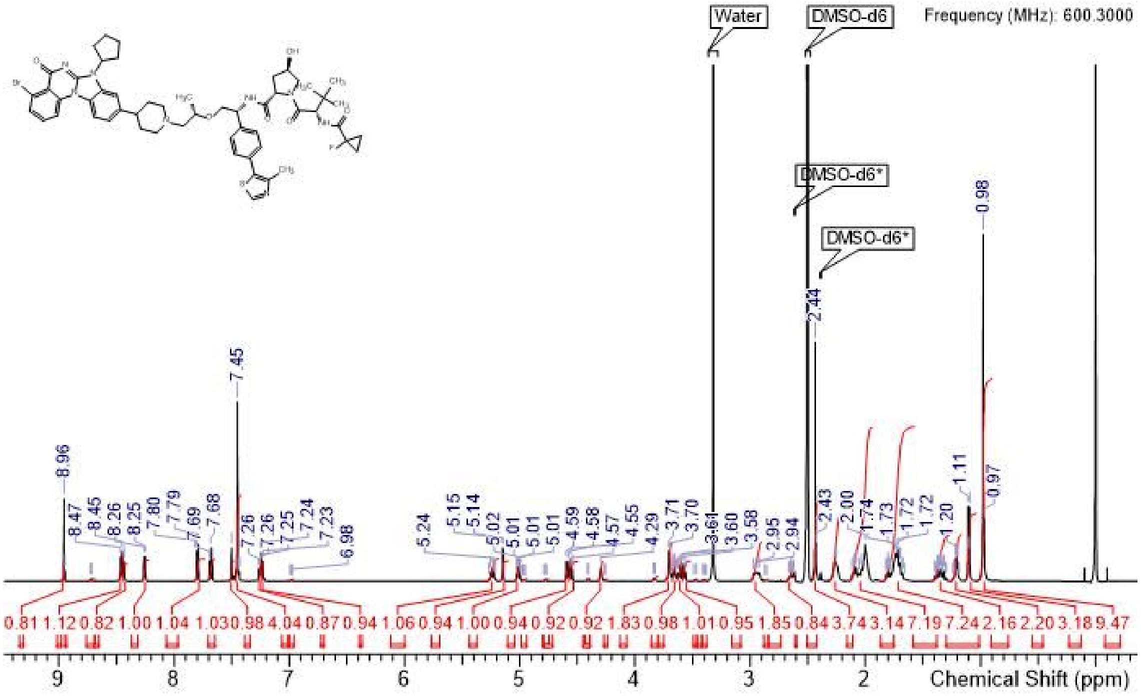
Water
DMSO-d6
Frequency (MHz): 600.3000
DMSO-d6*
DMSO-d6*
Chemical Shift (ppm)

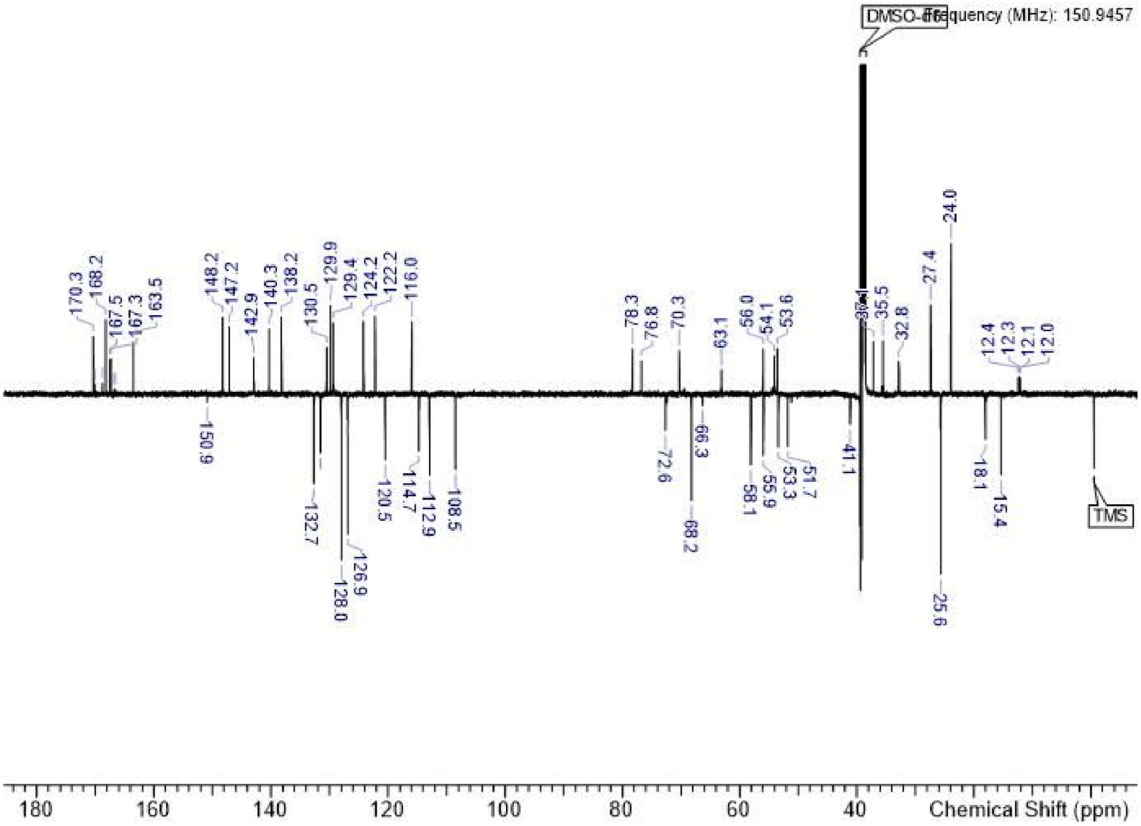
DMSO-d6
Frequency (MHz): 150.9457
TMS
Chemical Shift (ppm)

HPLC traces

A5-1

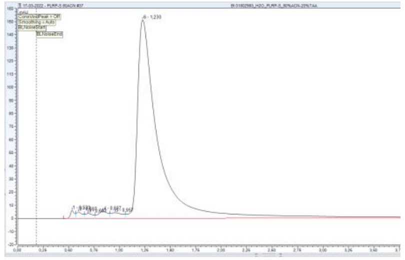

A5-2

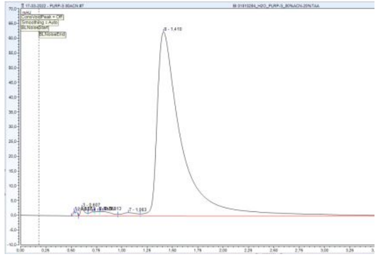

A5-3

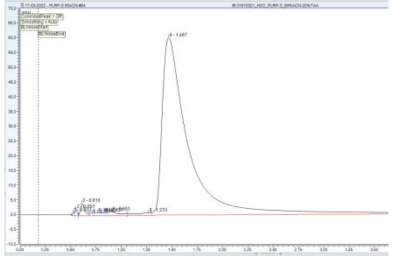

A5-4

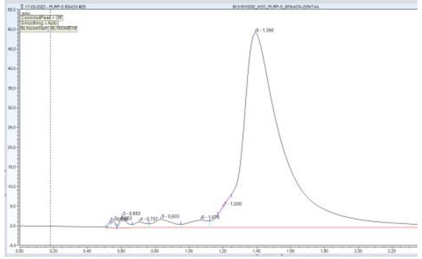

A3-1

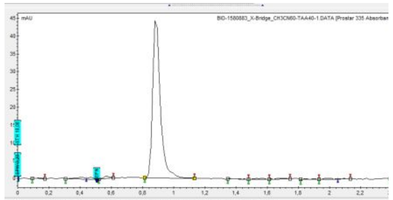

A3-2

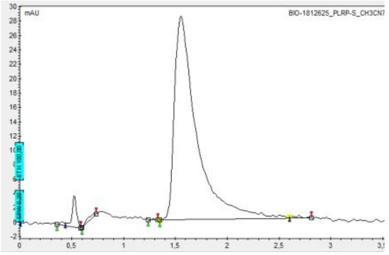

A3-3

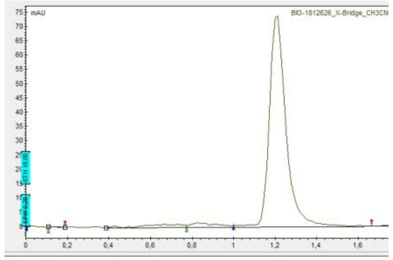

B-1

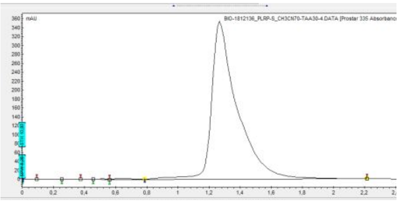

B-2

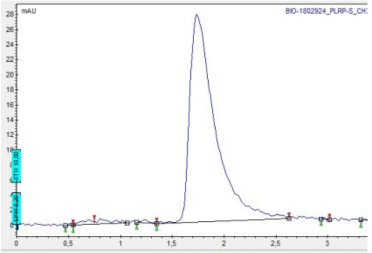

B-3

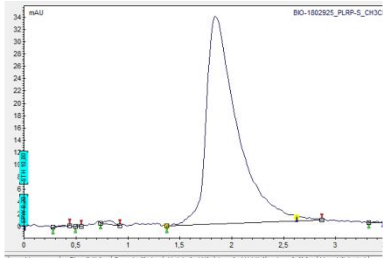

B-4

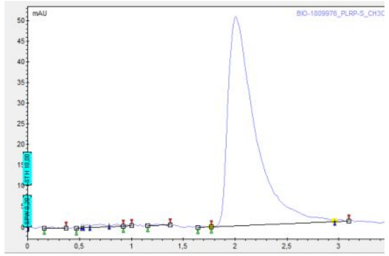

Supplement: Supplementary file 1 [file jm5c01497_si_001.pdf]
